# Supplementary material for: Factors associated with insufficient awareness of breast cancer among women in Northern and Eastern China: a case–control study
Source: BMJ Open. 2018 Feb 20;8(2):e018523. doi: 10.1136/bmjopen-2017-018523 (PMC5855304; doi:10.1136/bmjopen-2017-018523)
Supplement: Author's manuscript [file bmjopen-2017-018523.draft_revisions.pdf]

BMJ Open

BMJ Open is committed to open peer review. As part of this commitment we make the peer review history of every article we publish publicly available.

When an article is published we post the peer reviewers' comments and the authors' responses online. We also post the versions of the paper that were used during peer review. These are the versions that the peer review comments apply to.

The versions of the paper that follow are the versions that were submitted during the peer review process. They are not the versions of record or the final published versions. They should not be cited or distributed as the published version of this manuscript.

BMJ Open is an open access journal and the full, final, typeset and author-corrected version of record of the manuscript is available on our site with no access controls, subscription charges or pay-per-view fees (http://bmjopen.bmj.com).

If you have any questions on BMJ Open's open peer review process please email editorial.bmjopen@bmj.com

BMJ Open

Hospital-based study of Breast Cancer Awareness among Women in Northern and Eastern China

Journal:	*BMJ Open*
Manuscript ID	bmjopen-2017-018523
Article Type:	Research
Date Submitted by the Author:	07-Jul-2017
Complete List of Authors:	Liu, Li-Yuan; the Second Hospital of Shandong University, breast surgery<br>Wang, Yong-Jiu; the Second Hospital of Shandong University, breast surgery<br>Wang, Fei; the Second Hospital of Shandong University, breast surgery<br>Yu, Li-Xiang; the Second Hospital of Shandong University, breast surgery<br>Xiang, Yu-Juan; the Second Hospital of Shandong University, breast surgery<br>Zhou, Fei; the Second Hospital of Shandong University, breast surgery<br>Li, Liang; the Second Hospital of Shandong University, breast surgery<br>Zhang, Qiang; the Second Hospital of Shandong University, breast surgery<br>Fu, Qin-Ye; the Second Hospital of Shandong University, breast surgery<br>Ma, Zhong-Bing; the Second Hospital of Shandong University, breast surgery<br>Gao, De-Zong; the Second Hospital of Shandong University, breast surgery<br>Li, Yu-Yang; the Second Hospital of Shandong University, breast surgery<br>Yu, Zhi-Gang; the Second Hospital of Shandong University, breast surgery
**Primary Subject Heading**:	Public health
Secondary Subject Heading:	Oncology
Keywords:	Breast cancer, Awareness, Knowledge, Chinese women

SCHOLARONE™
Manuscripts

Hospital-based study of Breast Cancer Awareness among Women in Northern and Eastern China

Li-Yuan Liu[1], Yong-Jiu Wang[1], Fei Wang[1], Li-Xiang Yu[1], Yu-Juan Xiang[1], Fei Zhou[1], Liang Li[1], Qiang Zhang[1], Qin-Ye Fu[1], Zhong-Bing Ma[1], De-Zong Gao[1], Yu-Yang Li[1], Zhi-Gang Yu[1]

[1] Department of Breast Surgery, the Second Hospital of Shandong University, Jinan, China

E-mail: Li-Yuan Liu: liuliyuanforever@163.com, Yong-Jiu Wang: wyj737@163.com, Fei Wang: wangfei4@medmail.com.cn, Li-Xiang Yu: ylx@medmail.com.cn, Yu-Juan Xiang: xxiangyujuan@126.com, Fei Zhou: zflywin@163.com, Liang Li: liliang19772004@sina.com, Qiang Zhang: vomiting@medmail.com.cn, Qin-Ye Fu: fqyhome@163.com, Zhong-Bing Ma: mazbing@163.com, De-Zong Gao: gaohill100@yeah.net, Yu-Yang Li: jinaner@tom.com, Zhi-Gang Yu*: yzg@medmail.com.cn

*Dr. Prof. Zhi-Gang Yu, MD. Department of Breast Surgery, the Second Hospital of Shandong University. Jinan 250033, Shandong, China. Tel: +8613864182636 Fax: +86-531-88960949. E-mail: yzg@medmail.com.cn

Word count: 3304

For peer review only - http://bmjopen.bmj.com/site/about/guidelines.xhtml

ABSTRACT

Objectives: To investigate the awareness and level of knowledge of breast cancer among women in China.

Design: Case control study

Settings: This study was based on the database of the minister-affiliated hospital key project of the Ministry of Health of the People's Republic of China that included 21 hospitals in China between April 2012 and April 2013.

Participants: Matched study was designed among 2978 participants with Han ethinic aged between 25 and 70 years old.

Primary and secondary outcome measures: Student's t-test, Pearson's $\chi 2$ test, reliability analysis, exploratory factor analysis, and univariate and multivariate logistic regression analyses were performed to know the level of breast cancer knowledge and find out the factors associated with breast cancer awareness.

Results: 80.0% of Chinese women had poor awareness of breast cancer. In-depth knowledge of breast cancer, such as early symptoms and risk factors, was poor among these women. TV broadcast and relatives or friends with breast cancer were the main sources of information about breast cancer. Of all participants, 72.8% of women had heard about breast cancer as a frequent cancer affecting women, and 63.3% knew that family history of breast cancer was a risk factor for breast cancer. More than half of the women were aware that a breast lump could be a symptom of breast cancer. Multivariate logistic regression analysis identified the following variables that predicted awareness of breast cancer: age (OR=0.843), occupation (OR = 12.831), household social status (OR = 0.644), history of breast hyperplasia (OR =

For peer review only - http://bmjopen.bmj.com/site/about/guidelines.xhtml

1.684), and behavioral prevention score (OR = 4.407).

Conclusion: Most women were aware of breast cancer as a disease, but their in-depth knowledge of breast cancer was still poor. Increased publicity and education programs to increase breast cancer awareness are necessary and urgent, especially targeting aging women and agricultural workers.

Keywords: Breast cancer, Awareness, Knowledge, Chinese women

Strengths and limitations of this study

- This was a multicenter, matched case-control study designed to investigate the breast cancer awareness of women in 21 hospitals in Northern and Eastern China.
- We designed this hospital-based study to investigate the level of knowledge of breast cancer in female breast cancer patients. Meanwhile, we compared the results with our previous community-based study.
- Although we used the same questionnaire in these two studies, we still did not have uniform standards and methods for measuring knowledge evaluation. Thence a standard measurement of breast cancer-related knowledge should be developed.

For peer review only

INTRODUCTION

Breast cancer (BC) is one of the most common cancers and the leading cause of cancer-related death among women worldwide [1]. Although in the past, China had a relatively low incidence of BC, recently BC incidence has been increasing much faster in China than globally [2]. According to latest statistics from the National Central Cancer Registry, breast cancer is the most frequently diagnosed cancer among Chinese women in all age and ethnic groups, accounting for nearly one fifth of all cancer types. Moreover, by 2011, BC incidence had increased to 32.43/100,000, which is higher than the average BC incidence in East Asia (27/100,000) [3].

Many studies have shown that the early detection of BC plays a vital role in patient survival. Further, a delay in the diagnosis and subsequent treatment can lead to worsening of morbidity and mortality [4, 5]. From the time of onset to that of diagnosis, patients may experience disease progression, which could lead to tumor growth, and consequently, worse outcomes [6]. Stages at diagnosis differ among countries with different incomes. A report showed that more than 70% of women with BC in developed countries had disease stage I or II, compared with 20% to 60% in low and middle-income countries [7]. A commercial report showed that in China, nearly two-thirds of BC patients were diagnosed with advanced disease, which was obviously higher than that in the USA [2]. It is well-known that BC screening is an effective way to detect early-stage disease; however, in contrast to developed countries, China has no such current nationwide BC screening program because of economic and demographic factors [8]. Additionally, people are not considered to consciously have the appropriate attitudes toward such BC screening programs [9]. Many studies have shown that the level of cancer

For peer review only - http://bmjopen.bmj.com/site/about/guidelines.xhtml

awareness is a significant risk factor for early detection of BC [9-11]. Therefore, it is necessary to implement interventions aimed at increasing the comprehensive knowledge and awareness of BC symptoms and screening methods.

To promote BC awareness among Chinese women and build education programs to prevent delays in diagnosis and treatment, healthcare specialists must know their current level of understanding. Thus, we performed this cross-sectional survey in China to assess the level of awareness and knowledge of breast cancer-related symptoms and risk factors, and identify awareness-related factors.

METHODS

Study design

We designed a multicenter, case-control hospital-based study to investigate the awareness of women in 21 hospitals located in 11 provinces in Northern and Eastern China. This study was conducted between April 2012 and April 2013 and was funded by the Ministry of Health of the People's Republic of China.

Study population

All participants were of the Han ethnic group. Cases and controls were matched 1:1 for age (±3 years) and timing of examination (within 2 months). The inclusion criteria for BC group were as follows: (1) newly diagnosed and histologically confirmed BC and (2) women aged between 25 and 70 years old. Women who had recurrent or metastatic BC and/or complications of other malignant tumors confirmed by clinical or pathological diagnosis were excluded from this group. The inclusion criteria for the control group were as follows: (1) negative physical examination results; (2) negative ultrasound scans of breast and/or

For peer review only - http://bmjopen.bmj.com/site/about/guidelines.xhtml

mammographic screening results; and (3) no evidence or history of cancer. The exclusion criteria for the control group were as follows: a neoplastic disease at any other site, history of cancer or other major chronic diseases. We collected data strictly according to the inclusion and exclusion criteria, and then excluded subjects whose data were incomplete or lacking. Finally, a total of 1489 case-control sets were involved.

Data collection

A self-designed structured questionnaire was previously developed to record information through person-to-person interviews. The theoretical bases of this interview questionnaire were numerous published articles and the opinions of a variety of experts in breast surgery, epidemiology, statistics, nutrition, and molecular biology. Several similar questions were asked in different sections of the questionnaire to minimize recall bias. Previously, we conducted an investigation to assess the practicality and effectiveness of the survey and by which we validated the questionnaire[8]. The final interviewer-administered questionnaire was composed of the following parts: (1) demographic characteristics, physiological and reproductive factors, such as current age, age at menarche, age at menopause, menopausal status; (2) chronic diseases and family history such as breast hyperplasia, diabetes mellitus, hypertension and family history of BC; (3) lifestyle habits: smoking, alcohol intake, dietary habits, sleeping satisfaction; (4) awareness of breast cancer-related knowledge: sources of breast cancer-related information, risk factors and early signs or symptoms of BC (cumulative scores of these relevant items were counted as the related knowledge score and behavioral prevention score); (5) medical records including the visual examination, palpation and related diagnostic tests results. Additionally, for BC patients, the histological and

For peer review only - http://bmjopen.bmj.com/site/about/guidelines.xhtml

For peer review only

immunohistochemical diagnosis were also evaluated.

Scoring scheme

Awareness and knowledge of BC were assessed through 15 items on risk factors and early symptoms of BC included in the questionnaire (Table 2). For each item, if respondents gave a correct response (“yes”), they scored one (1) point; if a wrong response (“no” or “do not know”) was given, the score for the item was zero (0). Total scores thus ranged from 0 to 15. Then, we set a score to identify the status of respondents’ awareness and knowledge of BC. Respondents with scores ranging from 0 to 8 were considered to have poor awareness and knowledge, whereas those with scores ranging from 9 to 15 points were considered to have high awareness and knowledge. Behavioral prevention was scored cumulatively by five items: participation in BC screening, breast self-examination (BSE), clinical breast examination (CBE), radiographic breast examination, and breast ultrasound examination. Its scoring rules were the same as the 15-item questionnaire, and the total scores ranged from 0 to 5. The overall life satisfaction score was cumulatively based on 12 items; high scores meant low life satisfaction, and low scores indicated high life satisfaction.

Quality control

Interviewers were selected by medical professionals and medical post-graduate students. All interviewers had completed standardized training and were certified to conduct surveys independently. The questionnaires and forms were coded twice and were entered twice by different clerks. If there were inconsistent records, professionals would manually check and correct these. We also used computer software to check the logic and reasonable range of responses throughout the questionnaire to identify contradictory responses.

For peer review only - http://bmjopen.bmj.com/site/about/guidelines.xhtml

Statistical analysis

EpiData3.1 was used to create the database. Statistical methods, including Student's t-test, Pearson's $\chi2$ test, reliability analyses, exploratory factor analysis, and univariate and multivariate logistic regression analyses, were used to identify factors related to the knowledge of BC. Odds ratios (OR) with 95% confidence intervals were also calculated. All data analyses were performed using SPSS21.0.

RESULTS

In this survey, 2978 women were all included in our final analysis. The demographic characteristics are shown in Table 1. The mean age of participants was 47.38±8.8 years. A total of 1611 subjects (54.1%) were from urban areas and 1248 (41.9%) were from rural areas. More than one-third of the population (33.9%) consisted of agricultural workers. Nearly half of the women (1422, 47.8%) had other occupations; some were teachers, drivers, or provided civil service; 441 (14.8%) were workers; and 106 (3.6%) were medical staff. Regarding subject distribution based on education status, 466 subjects (15.6%) had primary school studies or less, 887 (29.8%) had completed middle school, 914 (30.7%) had completed high school, 565 (19.0%) had college degrees and 36 (1.2%) had post-graduate degrees. Most of the responders (2810, 94.4%) were married, while 33 (1.1%) were single and 135 (4.5%) were widowed or divorced. In terms of income level, 140(4.7%) earned less than 1000 Chinese Yuan Renminbi (RMB) per month, 394 (13.2%) earned 1000–1999 RMB monthly, a quarter (24.9%) of the subjects had a monthly family income of 2000–2999 RMB, 791(26.6%) of the women had monthly family income of 3000–4999 RMB per month and 821 (27.6%) subjects had a monthly family income greater than 5000 RMB.

For peer review only - http://bmjopen.bmj.com/site/about/guidelines.xhtml

Table 1 Main characteristics of responders (N=2,978)

Category		Awarenes N		χ2	*p*
		high	low		
**Present history of breast cancer**					
	yes	248	1241	20.585	＜0.001
	no	347	1142		
**Age group（years）**					
	25-	56	149		
	35-	214	723		
	45-	206	950	18.655	0.001
	55-	108	490		
	65-	11	71		
	mean（SD）	47.38（8.8）			
**Location**					
	urban	465	1146	189.36	＜0.001
	rural	102	1146		
**Marital status**					
	married	559	2251		
	single	10	23	2.257	0.324
	widowed/divorced	26	109		
**Monthly household income（RMB）**					
	＜1000	7	133	151.925	＜0.001

For peer review only - http://bmjopen.bmj.com/site/about/guidelines.xhtml

1000-1999	53	341		
2000-2999	111	631		
3000-4999	129	662		
≥5000	281	540		
**Occupation**				
agricultural workers	63	946		
worker	94	347	281.37	＜0.001
medical staff	68	38		
others	370	1052		
**Educational status**				
primary or less	26	440		
middle	122	765		
high	216	698	186.918	＜0.001
college	201	364		
post-graduate	16	20		
**Menopause**				
yes	175	760	1.520	0.218
no	400	1534		
**History of breast hyperplasia**				
yes	200	388	91.447	＜0.001
no	383	1954		
**Family history of breast cancer**				

For peer review only - http://bmjopen.bmj.com/site/about/guidelines.xhtml

	yes	47	98	14.228	＜0.001
	no	528	2178		
**Smoking**					
	yes	7	76	7.100	0.008
	no	585	2298		
**Drinking**					
	yes	106	290	12.908	＜0.001
	no	488	2080		
**BMI**					
	≤23.9	290	1105		
	24.0-27.9	220	868	1.649	0.438
	≥28.0	58	271		
**WHR**					
	＜0.85	288	919	17.609	＜0.001
	≥0.85	219	1063		
**Present life satisfaction**					
	high	332	1492	9.308	0.002
	low	263	891		
**Behavioral prevention score**					
	low	263	891	9.308	0.002
	high	332	1492		

For peer review only

For peer review only - http://bmjopen.bmj.com/site/about/guidelines.xhtml

Responses to questions about BC among the population are summarized in Table 2. A total of 2167 subjects (72.8%) had heard about BC as a common disease affecting women, 2253 (75.7%) thought BC screening was helpful for early detection of BC, and 2386 (80.1%) believed that early detection of BC could improve survival. In terms of the knowledge of risk factors for BC, 1884 (63.3%) knew that family history of BC was a risk factor for BC; and 381 (12.8%) knew that women whose menarche age was less than 12 years are at higher risk of developing BC; 679 (22.8%) knew that no parity and late childbirth increased the risk of BC, 495 (16.6%) knew that late menopause was associated with BC, 636 (21.4%) considered high-fat diet as a risk factor, 616 (20.7%) knew that long-term drinking habits increased BC risk and 937 (31.5%) knew that long-term use of estrogen drugs was also a risk factor for BC. Regarding the awareness of early symptoms of BC, more than half (52.7%) of the subjects were aware that a breast lump could be a symptom of BC. Awareness of other symptoms was higher when compared with our previous community-based survey results (Figure 1). In summary, 595 women (20.0%) showed high BC awareness and 2383 (80.0%) poor BC awareness.

Table 2 Responses from the 15-item questionnaire for breast cancer awareness (N=2,978)

Questions	N	%
**Do you know breast cancer**		
Yes	2167	72.8
No	736	24.7
**Do you think screening is helpful for early detection of breast cancer**		
Yes	2253	75.7

For peer review only - http://bmjopen.bmj.com/site/about/guidelines.xhtml

No	668	22.4
**Do you think the early detection of breast cancer can improve survival**		
Yes	2386	80.1
No	536	18.0
Knowledge about breast symptom		
**Local discomfort in breast**		
Yes	1107	37.2
No	657	22.1
Don't know	1176	39.5
**Lump in breast**		
Yes	1569	52.7
No	418	14.0
Don't know	953	32.0
**Axillary nodes**		
Yes	1001	33.6
No	369	12.4
Don't know	1561	52.4
**Nipple retraction**		
Yes	790	26.5
No	367	12.3
Don't know	1691	56.8

For peer review only

For peer review only - http://bmjopen.bmj.com/site/about/guidelines.xhtml

**Nipple discharge liquid**		
Yes	875	29.4
No	367	12.3
Don't know	1691	56.8
Related factors of breast cancer		
**Menarche at age before 12**		
Yes	381	12.8
No	471	15.8
Don't know	2088	70.1
**No parity or late childbirth**		
Yes	679	22.8
No	377	12.7
Don't know	1880	63.1
**Menopause at a late age**		
Yes	495	16.6
No	380	12.8
Don't know	2062	69.2
**Long time drinking**		
Yes	616	20.7
No	369	12.4
Don't know	1947	65.4
**High-fat diets**		

For peer review only - http://bmjopen.bmj.com/site/about/guidelines.xhtml

Yes	636	21.4
No	353	11.9
Don't know	1947	65.4
**Long-term use of estrogen drugs**		
Yes	937	31.5
No	244	8.2
Don't know	1752	58.8
**Family history of breast cancer**		
Yes	1884	63.3
No	158	5.3
Don't know	895	30.1
**Awareness of breast cancer**		
Highly aware	595	20.0
Poorly aware	2383	80.0

Regarding the subjects' BC behavioral prevention, 1109 (37.2%) women had performed BSE at least once, 1020 of the 2978 women (37.2%) had undergone clinical breast examination, 35.9% had received breast ultrasound and 22.2% had undergone a radiographic breast examination. We set a 3-point cut-off value to distinguish high and low awareness of breast examination, and we found that more than half of the women (1824, 61.2%) had high awareness of behavioral prevention.

Most of the subjects obtained their information on BC from television broadcast and relatives or friends with BC, which accounted for 30.6% and 29.6% of all women, respectively (Figure

For peer review only - http://bmjopen.bmj.com/site/about/guidelines.xhtml

2).

Results for univariate analysis indicated that awareness and knowledge of BC were related to history of BC, age, location, education status, occupation, household monthly income, social status, history of breast hyperplasia, family history of BC, drinking, smoking, physical training execution, waist-hip ratio, present life satisfaction, and behavioral prevention score. Multivariate analysis identified that age (OR: 0.843 95.0%CI: 0.740-0.961), occupation (agricultural worker vs. worker, medical staff: OR=3.066, 95% CI: 1.999–4.703; OR=12.831, 95% CI: 6.998–23.523, respectively), social status (OR: 0.644, 95%CI: 0.531–0.780), history of breast hyperplasia (OR: 1.684, 95%CI: 1.273–2.228), and behavioral prevention score (OR: 4.407, 95%CI: 3.433–5.657) were independently correlated with BC awareness and knowledge (Table 3).

Table 3 Multivariate analysis of factors related to knowledge of breast cancer

	B	*S.E.*	*Wald*	*p*	*OR*	*95.0%CI*
Age	-0.170	0.067	6.511	0.011	0.843	0.740-0.961
Occupation						
agricultural worker				<0.001	1.000*	
worker	1.120	0.218	26.335	<0.001	3.066	1.999-4.703
medical staff	2.552	0.309	68.078	<0.001	12.831	6.998-23.523
other	1.238	0.183	45.721	<0.001	3.448	2.408-4.936
Household social status	-0.441	0.098	20.292	<0.001	0.644	0.531-0.780
History of breast hyperplasia	0.521	0.143	13.298	<0.001	1.684	1.273-2.228

For peer review only - http://bmjopen.bmj.com/site/about/guidelines.xhtml

Behavioral prevention score	1.483	0.127	135.509	<0.001	4.407	3.433-5.657

* Reference.

Occupation: "Other" includes occupations such as teacher, civil servant, individual business, driver, service, company employee, and housewife.

Household social status, reference: low social status.

History of breast hyperplasia, reference: absence of breast hyperplasia history.

Behavioral prevention score was a cumulative score of 5 items; reference: low scores.

RMB, Chinese Yuan Renminbi; BMI, body mass index; WHR, waist-hip ratio

Reliability and construct validity and internal consistency reliability estimates of the 15-item scale of awareness and knowledge of BC were calculated using Cronbach's alpha. The α-coefficient for the total scale was 0.902, which was considered acceptable for internal consistency reliability [12]. Exploratory factor analysis was conducted to explore construct validity. The Kaiser–Meyer–Olkin measure produced a coefficient of 0.883, indicative of excellent sampling adequacy. Bartlett's test of sphericity produced a value of 23825.328 ($p <$ 0.001), indicating that the correlation matrix was unlikely to be an identity matrix and was therefore suitable for factor analysis [13].

DISCUSSION

In this hospital-based study, we evaluated the level of BC awareness among Chinese women. Results showed that most participants had poor awareness regarding BC (80.0%), which is similar to our previous community-based study (81.4% of subjects had poor awareness). This seems to be a common phenomenon both in developing and developed countries, although the proportion of women with high awareness in developed countries was reportedly higher than that in developing countries or regions [14, 15].

It has also been reported that different approaches for obtaining cancer-related knowledge influenced the level of awareness of BC [16]. We investigated the resources by which women obtained knowledge of BC, and we found that the majority of women obtained BC

For peer review only - http://bmjopen.bmj.com/site/about/guidelines.xhtml

information through traditional media such as TV broadcasts (30.6%) and their friends or relatives with BC (29.6%). The Internet has developed rapidly and become widespread, but in the present study, women failed to benefit from it in terms of BC awareness (8.6%). Less women participated in special lectures about BC (2.5%), which indicated that the effort to publicize such events for BC were likely insufficient. A study comparing non-Hispanic and Hispanic college women revealed that the Internet was the most common information resource (75%) [17], while a Spanish survey indicated that the main sources of information were television, press, family, and friends [18]. In Malaysia and Cameroon, television was still the major resource for obtaining knowledge [19, 20]. It was worth noting that an increasing number of people are using the Internet to obtain information on diseases such as cancer. However, in our country, women were not accustomed to employing the Internet to search for cancer-related information, which may be a possible consequence of cultural and economic diversity.

In our study, more than two-thirds (72.8%) of the women knew or had heard about BC, but their in-depth knowledge of the early symptoms of BC and risk factors was insufficient. Although more than half (52.7%) of the women knew that the presence of a lump in the breast was a BC symptom and family history was an important risk factor for BC, the proportion of women who knew other BC symptoms and BC risk factors was low (overall, less than 40%). These results were consistent with our previous study [8] (Figure 1), which indicated that although most women in China knew or had heard about BC, their in-depth knowledge of BC needed to be urgently improved. Studies from other countries showed consistent results [4, 14, 21, 22], especially those conducted in developing countries. In developed countries, the proportion

For peer review only - http://bmjopen.bmj.com/site/about/guidelines.xhtml

of women reporting cancer-related symptoms was higher to a certain extent; this may be a possible consequence of their higher living standards, a greater consciousness of health, and more social publicity.

Many studies found a close relationship between age and awareness and knowledge of BC [9, 21, 23, 24]. The results of a study by Mandelblatt J et al. revealed that the level of knowledge of BC decreased as the age of responders increased [25]. Several articles researching Indian women also yielded similar results [9, 26]. However, a study completed by Sen et al. showed that older women were more interested in BC knowledge than younger women. Okobia et al. also arrived at the same conclusion among Nigerian women [27]. In our study, we found that age was related to BC awareness as well (OR=0.843, 95% CI: 0.740–0.961). Younger women tended to have more awareness and knowledge of BC although older women were at higher risk of BC development. Thus, it is urgent to improve the awareness and knowledge of older women to decrease the incidence of malignant breast tumors in this population.

The relationship between occupation and the level of awareness and knowledge on BC were also demonstrated in this study. Workers and medical staff tended to be more aware of the symptoms and risk factors for BC, while the awareness of agricultural workers was significantly poorer. Similar results were observed in many other studies including our community-based population survey [8, 28, 29]. Nonetheless, an Iranian study showed that the awareness of BC of rural women was moderate; this may indicate the existence of ethnic and population differences [10]. As is shown in both community- and hospital-based studies, medical personnel were more aware of BC than agricultural workers (OR: 4.774, 95% CI: 4.316-5.281), likely benefiting from easier access to relevant knowledge.

For peer review only - http://bmjopen.bmj.com/site/about/guidelines.xhtml

Many studies revealed that education level was a major determinant of BC awareness, increased awareness was associated with higher levels of education. Terzioglu [30] stated that the awareness of BC was attributed to the respondents' education level. This was also stated in the study by Manas Kotepui [31]. The univariate analysis in our study consistently showed that awareness and knowledge of BC were related to education status; however, the result was not shown in our multivariate analysis. The study by Cannas Kwok [32] had a similar conclusion among Arabic Australia women, in whom demographic characteristics, such as education level, were a negative factor. Education level was associated with several other factors such as occupation, income, among others, but further research is warranted to clarify other factors involved.

Women with a history of breast hyperplasia tended to know more about BC in our study (OR: 1.684, 95%CI: 1.273–2.228). In some cases, the symptoms of breast hyperplasia may be similar to those of cancer; thus, women who had been diagnosed with breast hyperplasia were likely to pay more attention to breast health and to take better precautions. Breast hyperplasia may cause breast tenderness and other symptoms, but it may also serve as a forewarning for increased self-health awareness. This result was consistent with the research by Kosgeroglu et al. [33], in which it was shown that if women were diagnosed previously with benign breast diseases, they would make efforts to obtain sufficient knowledge of BC.

Regarding behavioral prevention of BC, our study showed that 61.2% of women adequately and consciously performed breast examination. Among the women with high awareness of BC, most had accepted breast-related examinations and 61.2% of them obtained a high behavioral prevention score. However, the proportions of women who accepted BSE, CBE,

For peer review only - http://bmjopen.bmj.com/site/about/guidelines.xhtml

breast ultrasound or breast radiograph were still low (less than 40%). Rates of BSE practices, clinical examination, and radiographic screening were lower than those reported in the studies of Whitman et al. [34] and C Kwok, et al. [35] (55.3% and 90% had a mammogram in Chicago and Australia, respectively). The cause for this difference may be the lack of related knowledge and insufficient guidance of treatment. The relationship between early detection and BSE remains controversial, but most scholars still believe that self-examination probably improves awareness and might play an important role in nationwide programs for earlier-stage detection in China. From this point of view, breast examination is helpful for early diagnosis and decreased mortality.

In this study, the result of the reliability index was 0.902 and the validity was 0.883, while the results in our previous study were 0.910 and 0.870, respectively. We used the same questionnaire in these two studies, but we still did not have uniform standards and methods for measuring breast cancer-related knowledge evaluation. Thus, we consider that a standard measurement of breast cancer-related knowledge should be developed. Additionally, the reality and validity of our 5-items questionnaire for assessing levels of behavioral prevention was not good enough for this assessment (Cronbach's alpha 0.769 and KMO coefficient 0.780), which limited the results of the study in terms of BC practices.

CONCLUSIONS

Combined with our previous study, we concluded that most women were aware of BC as a disease entity, but their in-depth knowledge of the disease was poor. BC awareness, increased publicity, and education programs are necessary and urgent, especially for older women and agricultural workers.

For peer review only - http://bmjopen.bmj.com/site/about/guidelines.xhtml

Acknowledgments We would like to thank all of the subjects involved in the study for their participation.

Contributors: ZGY and LYL conceived and designed the interviews; LXY, FW, LL, QZ, YYL, DZG and QYF performed the interviews; YJW, YJX and LYL analyzed the data; FZ and ZBM prepared the tables and figures; LYL, YJW and FW wrote the paper; ZGY supplied suggestions**.**

Funding This research was funded by the minister-affiliated hospital key project of the Ministry of Health of the People's Republic of China (Establishment and improvement of high-risk populations screening and evaluation system for breast cancer) and the project of the Second Hospital of Shandong University (Research of risk assessment model building based on the multiple health data fusion).

Competing interests The authors declare that they have no competing interests.

Ethics approval The study protocol and procedures were approved by the Institutional Review Board at the Second Hospital of Shandong University. Before conducting the interviews and under the investigators' guidance, all participants provided written informed consent.

For peer review only

For peer review only - http://bmjopen.bmj.com/site/about/guidelines.xhtml

References

1. DeSantis CE, Bray F, Ferlay J, Lortet-Tieulent J, Anderson BO, Jemal A. International Variation in Female Breast Cancer Incidence and Mortality Rates. Cancer Epidemiol Biomarkers Prev. 2015. 24(10): 1495-506.

2. Fan L, Strasser-Weippl K, Li JJ, et al. Breast cancer in China. Lancet Oncol. 2014. 15(7): e279-89.

3. GLOBOCAN: Estimated Cancer Incidence, Mortality and Prevalence Worldwide; 2012. http://globocan.iarc.fr/Default.aspx Accessed 17 March 2014.

4. Gupta A, Shridhar K, Dhillon PK. A review of breast cancer awareness among women in India: Cancer literate or awareness deficit. Eur J Cancer. 2015. 51(14): 2058-66.

5. Abu-Helalah AM, Alshraideh AH, Al-Hanaqtah M, Da'na M, Al-Omari A, Mubaidin R. Delay in Presentation, Diagnosis, and Treatment for Breast Cancer Patients in Jordan. Breast J. 2016. 22(2): 213-7.

6. Webber C, Jiang L, Grunfeld E, Groome PA. Identifying predictors of delayed diagnoses in symptomatic breast cancer: a scoping review. Eur J Cancer Care (Engl). 2016 .

7. Salih AM, Alfaki MM, Alam-Elhuda DM, Nouradyem MM. Factors Delaying Presentation of Sudanese Breast Cancer Patients: an Analysis Using Andersen's Model. Asian Pac J Cancer Prev. 2016. 17(4): 2105-10.

8. Liu LY, Wang F, Yu LX, et al. Breast cancer awareness among women in Eastern China: a cross-sectional study. BMC Public Health. 2014. 14: 1004.

9. Dey S, Mishra A, Govil J, Dhillon PK. Breast Cancer Awareness at the Community Level among Women in Delhi, India. Asian Pac J Cancer Prev. 2015. 16(13): 5243-51.

For peer review only - http://bmjopen.bmj.com/site/about/guidelines.xhtml

10. Balouchi A, Shahdadi H, AlKhasawneh E, et al. Rural Women's Awareness about Breast Cancer in Southeastern Iran: a Cross-Sectional Study. Asian Pac J Cancer Prev. 2016. 17(4): 1875-9.

11. Cruz-Castillo AB, Hernández-Valero MA, Hovick SR, Campuzano-González ME, Karam-Calderón MA, Bustamante-Montes LP. A Study on the Knowledge, Perception, and Use of Breast Cancer Screening Methods and Quality of Care Among Women from Central Mexico. J Cancer Educ. 2015. 30(3): 453-9.

12. Health measurement scales: a practical guide to their development and use (5th edition). Aust N Z J Public Health. 2016. 40(3): 294-5.

13. Tabachnick BG, Fidell LS: Using Multivariate Statistics. New York: Allyn & Bacon; 2001.

14. Forbes LJ, Atkins L, Thurnham A, Layburn J, Haste F, Ramirez AJ. Breast cancer awareness and barriers to symptomatic presentation among women from different ethnic groups in East London. Br J Cancer. 2011. 105(10): 1474-9.

15. Oluwatosin OA, Oladepo O: Knowledge of breast cancer and its early detection measures among rural women in Akinyele Local Government Area, Ibadan, Nigeria. BMC Cancer 2006, 6:271.

16. Lee EW, Shin M, Kawaja A, Ho SS. The Augmented Cognitive Mediation Model: Examining Antecedents of Factual and Structural Breast Cancer Knowledge Among Singaporean Women. J Health Commun. 2016. 21(5): 583-92.

17. Kratzke C, Amatya A, Vilchis H. Breast cancer prevention knowledge, beliefs, and information sources between non-Hispanic and Hispanic college women for risk reduction

For peer review only - http://bmjopen.bmj.com/site/about/guidelines.xhtml

focus. J Community Health. 2015. 40(1): 124-30.

18. Baena-Cañada JM, Rosado-Varela P, Expósito-Álvarez I, González-Guerrero M, Nieto-Vera J, Benítez-Rodríguez E. Women's perceptions of breast cancer screening. Spanish screening programme survey. Breast. 2014. 23(6): 883-8.

19. Akhtari-Zavare M, Ghanbari-Baghestan A, Latiff LA, Khaniki H. Breast cancer prevention information seeking behavior and interest on cell phone and text use: a cross-sectional study in Malaysia. Asian Pac J Cancer Prev. 2015. 16(4): 1337-41.

20. Nde FP, Assob JC, Kwenti TE, Njunda AL, Tainenbe TR. Knowledge, attitude and practice of breast self-examination among female undergraduate students in the University of Buea. BMC Res Notes. 2015. 8: 43.

21. Aydogan U, Doganer YC, Kilbas Z, et al. Predictors of knowledge level and awareness towards breast cancer among Turkish females. Asian Pac J Cancer Prev. 2015. 16(1): 275-82.

22. Khan TM, Leong JP, Ming LC, Khan AH. Association of Knowledge and Cultural Perceptions of Malaysian Women with Delay in Diagnosis and Treatment of Breast Cancer: a Systematic Review. Asian Pac J Cancer Prev. 2015. 16(13): 5349-57.

23. Grunfeld EA, Ramirez AJ, Hunter MS, Richards MA. Women's knowledge and beliefs regarding breast cancer. Br J Cancer. 2002. 86(9): 1373-8.

24. Linsell L, Burgess CC, Ramirez AJ. Breast cancer awareness among older women. Br J Cancer. 2008. 99(8): 1221-5.

25. Mandelblatt J, Kaufman E, Sheppard VB, et al. Breast cancer prevention in community clinics: will low-income Latina patients participate in clinical trials. Prev Med. 2005. 40(6): 611-8.

For peer review only - http://bmjopen.bmj.com/site/about/guidelines.xhtml

26. Shalini, Varghese D, Nayak M. Awareness and impact of education on breast self examination among college going girls. Indian J Palliat Care. 2011. 17(2): 150-4.

27. Okobia MN, Bunker CH, Okonofua FE, Osime U. Knowledge, attitude and practice of Nigerian women towards breast cancer: a cross-sectional study. World J Surg Oncol. 2006. 4: 11.

28. Santorelli ML, Hirshfield KM, Steinberg MB, Rhoads GG, Lin Y, Demissie K. Hormonal therapy for breast cancer and diabetes incidence among postmenopausal women. Ann Epidemiol. 2016 .

29. Mandelblatt JS, Huang K, Makgoeng SB, et al. Preliminary Development and Evaluation of an Algorithm to Identify Breast Cancer Chemotherapy Toxicities Using Electronic Medical Records and Administrative Data. J Oncol Pract. 2014 .

30. Terzioğlu, Özgü E, Kılıç, Yıldız, Güngör T. Evaluation of Breast Cancer Knowledge and Awareness Among Hospital Staff in a Women Heath Hospital in Turkey. J Cancer Educ. 2016 .

31. Kotepui M, Piwkham D, Chupeerach C, Duangmano S. Knowledge, attitudes and practice of breast cancer screening among female personnel of Walailak University. Health Expect. 2015. 18(6): 3069-78.

32. Kwok C;Endrawes G;Lee CF Cultural Beliefs and Attitudes About Breast Cancer and Screening Practices Among Arabic Women in Australia. Cancer Nurs.2016.39 (5):367-74.

33. Kosgeroglu N, Ayranci U, Ozerdogan N, et al (2011). Knowledge of women on early diagnosis methods and risk factors for breast cancer in a province of Western Turkey: A descriptive study. Pakistan J Med Sci, 27, 646-50.

For peer review only - http://bmjopen.bmj.com/site/about/guidelines.xhtml

34. Whitman S, Shah AM, Silva A, Ansell D. Mammography screening in six diverse communities in Chicago--a population study. Cancer Detect Prev. 2007. 31(2): 166-72.

35. Kwok C, Tranberg R, Lee FC. Breast cancer knowledge, attitudes and screening behaviors among Indian-Australian women. Eur J Oncol Nurs. 2015. 19(6): 701-6.

Figure 1 Comparison of proportions of women aware of breast cancer symptoms between a community-based and hospital-based studies

Figure 2 Proportions of sources of information of breast cancer for women

For peer review only

For peer review only - http://bmjopen.bmj.com/site/about/guidelines.xhtml

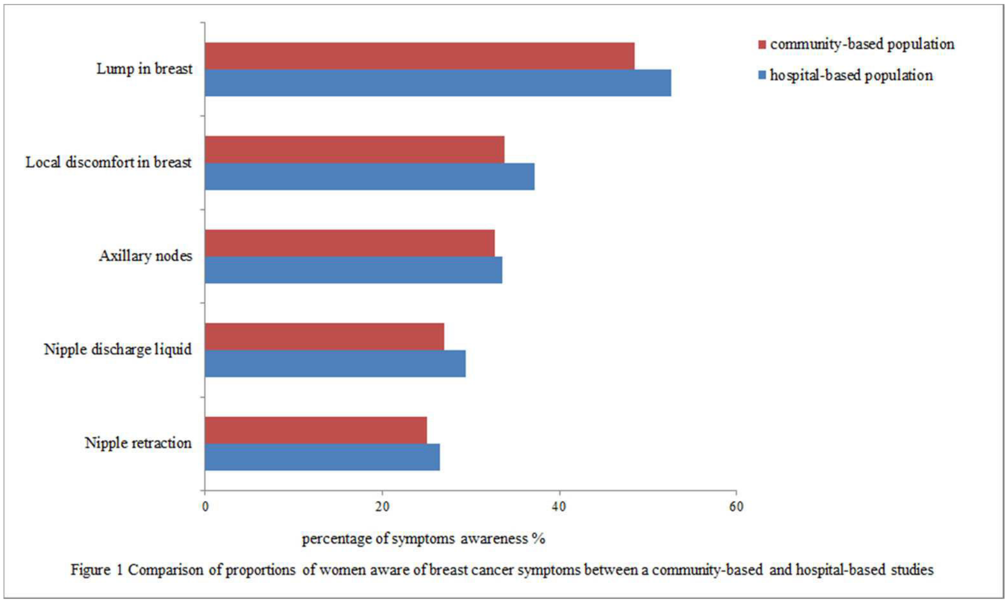

Figure 1 Comparison of proportions of women aware of breast cancer symptoms between a community-based and hospital-based studies

246x146mm (96 x 96 DPI)

For peer review only - http://bmjopen.bmj.com/site/about/guidelines.xhtml

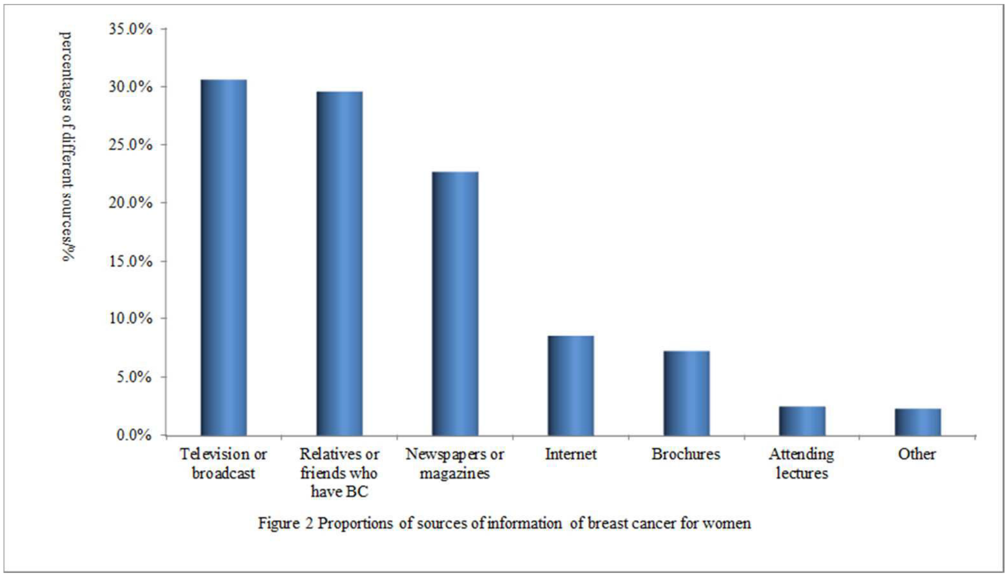

Figure 2 Proportions of sources of information of breast cancer for women

235x134mm (96 x 96 DPI)

STROBE Statement—Checklist of items that should be included in reports of ***case-control studies***

	Item No	Recommendation
**Title and abstract**	1	(*a*) Indicate the study's design with a commonly used term in the title or the abstract Page 2-3
		(*b*) Provide in the abstract an informative and balanced summary of what was done and what was found Page 2-3
**Introduction**		
Background/rationale	2	Explain the scientific background and rationale for the investigation being reported Page 4-5
Objectives	3	State specific objectives, including any prespecified hypotheses Page 5
**Methods**		
Study design	4	Present key elements of study design early in the paper Page 5
Setting	5	Describe the setting, locations, and relevant dates, including periods of recruitment, exposure, follow-up, and data collection Page 5-6
Participants	6	(*a*) Give the eligibility criteria, and the sources and methods of case ascertainment and control selection. Give the rationale for the choice of cases and controls Page 5-6
		(*b*) For matched studies, give matching criteria and the number of controls per case Page 5-6
Variables	7	Clearly define all outcomes, exposures, predictors, potential confounders, and effect modifiers. Give diagnostic criteria, if applicable Page 6-7
Data sources/ measurement	8*	For each variable of interest, give sources of data and details of methods of assessment (measurement). Describe comparability of assessment methods if there is more than one group Page 6-7
Bias	9	Describe any efforts to address potential sources of bias Page 7
Study size	10	Explain how the study size was arrived at Page 5
Quantitative variables	11	Explain how quantitative variables were handled in the analyses. If applicable, describe which groupings were chosen and why Page 6-7
Statistical methods	12	(*a*) Describe all statistical methods, including those used to control for confounding Page 8
		(*b*) Describe any methods used to examine subgroups and interactions Page 8
		(*c*) Explain how missing data were addressed Page 7
		(*d*) If applicable, explain how matching of cases and controls was addressed Page 5
		(*e*) Describe any sensitivity analyses Page 8
**Results**		
Participants	13*	(a) Report numbers of individuals at each stage of study—eg numbers potentially eligible, examined for eligibility, confirmed eligible, included in the study, completing follow-up, and analysed Page 8
		(b) Give reasons for non-participation at each stage
		(c) Consider use of a flow diagram
Descriptive data	14*	(a) Give characteristics of study participants (eg demographic, clinical, social) and information on exposures and potential confounders Page 8
		(b) Indicate number of participants with missing data for each variable of interest
Outcome data	15*	Report numbers in each exposure category, or summary measures of exposure Page8-11

For peer review only - http://bmjopen.bmj.com/site/about/guidelines.xhtml

Main results	16	(*a*) Give unadjusted estimates and, if applicable, confounder-adjusted estimates and their precision (eg, 95% confidence interval). Make clear which confounders were adjusted for and why they were included	Page 8-15
		(*b*) Report category boundaries when continuous variables were categorized	Page 8-15
		(*c*) If relevant, consider translating estimates of relative risk into absolute risk for a meaningful time period	Page 16-17
Other analyses	17	Report other analyses done—eg analyses of subgroups and interactions, and sensitivity analyses	
**Discussion**			
Key results	18	Summarise key results with reference to study objectives	Page 17-21
Limitations	19	Discuss limitations of the study, taking into account sources of potential bias or imprecision. Discuss both direction and magnitude of any potential bias	Page 17-21
Interpretation	20	Give a cautious overall interpretation of results considering objectives, limitations, multiplicity of analyses, results from similar studies, and other relevant evidence	Page 21
Generalisability	21	Discuss the generalisability (external validity) of the study results	Page 17
**Other information**			
Funding	22	Give the source of funding and the role of the funders for the present study and, if applicable, for the original study on which the present article is based	Page 22

*Give information separately for cases and controls.

Note: An Explanation and Elaboration article discusses each checklist item and gives methodological background and published examples of transparent reporting. The STROBE checklist is best used in conjunction with this article (freely available on the Web sites of PLoS Medicine at http://www.plosmedicine.org/, Annals of Internal Medicine at http://www.annals.org/, and Epidemiology at http://www.epidem.com/). Information on the STROBE Initiative is available at http://www.strobe-statement.org.

For peer review only

For peer review only - http://bmjopen.bmj.com/site/about/guidelines.xhtml

BMJ Open

Hospital-based study of Breast Cancer Awareness among Women in Northern and Eastern China

Journal:	*BMJ Open*
Manuscript ID	bmjopen-2017-018523.R1
Article Type:	Research
Date Submitted by the Author:	26-Oct-2017
Complete List of Authors:	Liu, Li-Yuan; the Second Hospital of Shandong University, breast surgery<br>Wang, Yong-Jiu; the Second Hospital of Shandong University, breast surgery<br>Wang, Fei; the Second Hospital of Shandong University, breast surgery<br>Yu, Li-Xiang; the Second Hospital of Shandong University, breast surgery<br>Xiang, Yu-Juan; the Second Hospital of Shandong University, breast surgery<br>Zhou, Fei; the Second Hospital of Shandong University, breast surgery<br>Li, Liang; the Second Hospital of Shandong University, breast surgery<br>Zhang, Qiang; the Second Hospital of Shandong University, breast surgery<br>Fu, Qin-Ye; the Second Hospital of Shandong University, breast surgery<br>Ma, Zhong-Bing; the Second Hospital of Shandong University, breast surgery<br>Gao, De-Zong; the Second Hospital of Shandong University, breast surgery<br>Li, Yu-Yang; the Second Hospital of Shandong University, breast surgery<br>Yu, Zhi-Gang; the Second Hospital of Shandong University, breast surgery
**Primary Subject Heading**:	Public health
Secondary Subject Heading:	Oncology
Keywords:	Breast cancer, Awareness, Knowledge, Chinese women

SCHOLARONE™ Manuscripts

Hospital-based study of Breast Cancer Awareness among Women in Northern and Eastern China

Li-Yuan Liu[1], Yong-Jiu Wang[1], Fei Wang[1], Li-Xiang Yu[1], Yu-Juan Xiang[1], Fei Zhou[1], Liang Li[1], Qiang Zhang[1], Qin-Ye Fu[1], Zhong-Bing Ma[1], De-Zong Gao[1], Yu-Yang Li[1], Zhi-Gang Yu[1]

[1] Department of Breast Surgery, the Second Hospital of Shandong University, Jinan, China

E-mail: Li-Yuan Liu: liuliyuanforever@163.com, Yong-Jiu Wang: wyj737@163.com, Fei Wang: wangfei4@medmail.com.cn, Li-Xiang Yu: ylx@medmail.com.cn, Yu-Juan Xiang: xxiangyujuan@126.com, Fei Zhou: zflywin@163.com, Liang Li: liliang19772004@sina.com, Qiang Zhang: vomiting@medmail.com.cn, Qin-Ye Fu: fqyhome@163.com, Zhong-Bing Ma: mazbing@163.com, De-Zong Gao: gaohill100@yeah.net, Yu-Yang Li: jinaner@tom.com, Zhi-Gang Yu*: yzg@medmail.com.cn

*Dr. Prof. Zhi-Gang Yu, MD. Department of Breast Surgery, the Second Hospital of Shandong University. Jinan 250033, Shandong, China. Tel: +8613864182636 Fax: +86-531-88960949. E-mail: yzg@medmail.com.cn

Word count: 4088

For peer review only - http://bmjopen.bmj.com/site/about/guidelines.xhtml

For peer review only

ABSTRACT

Objectives: To investigate the awareness and level of knowledge of breast cancer among women in China.

Design: Case control study

Settings: This study was based on the database of the minister-affiliated hospital key project of the Ministry of Health of the People's Republic of China that included 21 hospitals in China between April 2012 and April 2013.

Participants: Matched study was designed among 2978 participants with Han ethnicity aged between 25 and 70 years old.

Primary and secondary outcome measures: Student's t-test, Pearson's $\chi2$ test, reliability analysis, exploratory factor analysis, and univariate and multivariate logistic regression analyses were performed to know the level of breast cancer knowledge and find out the factors associated with breast cancer awareness.

Results: 80.0% of Chinese women had poor awareness level of breast cancer. In-depth knowledge of breast cancer, such as early symptoms and risk factors, was poor among these women. TV broadcast and relatives or friends with breast cancer were the main sources of information about breast cancer. Of all participants, 72.8% of women had heard about breast cancer as a frequent cancer affecting women, and 63.3% knew that family history of breast cancer was a risk factor for breast cancer. More than half of the women were aware that a breast lump could be a symptom of breast cancer. Multivariate logistic regression analysis identified the following variables that predicted awareness of breast cancer: young age (OR=0.843), occupation (agricultural worker) (OR = 12.831), high household social status

For peer review only - http://bmjopen.bmj.com/site/about/guidelines.xhtml

(OR = 0.644), history of breast hyperplasia (OR = 1.684), and high behavioral prevention score (OR = 4.407).

Conclusion: Most women were aware of breast cancer as a disease, but their in-depth knowledge of breast cancer was still poor. Increased publicity and education programs to increase breast cancer awareness are necessary and urgent, especially targeting aging women and agricultural workers.

Keywords: Breast cancer, Awareness, Knowledge, Chinese women

Strengths and limitations of this study

- This was a multicenter, matched case-control study designed to investigate the breast cancer awareness of women in 21 hospitals in Northern and Eastern China.
- We designed this hospital-based study to investigate the level of knowledge of breast cancer in female breast cancer patients. Meanwhile, we compared the results with our previous community-based study.
- Although we used the same questionnaire in these two studies, we still did not have uniform standards and methods for measuring knowledge evaluation. Thence a standard measurement of breast cancer-related knowledge should be developed.

For peer review only

INTRODUCTION

Breast cancer (BC) is one of the most common cancers and the leading cause of cancer-related death among women worldwide [1]. Although in the past, China had a relatively low incidence of BC, recently BC incidence has been increasing much faster in China than globally on account of change of diet, lifestyle and unique one-child policy [2, 3]. According to latest statistics from the National Central Cancer Registry, breast cancer is the most frequently diagnosed cancer among Chinese women in all age and ethnic groups, accounting for nearly one fifth of all cancer types. Moreover, by 2011, BC incidence had increased to 32.43/100,000, which is higher than the average BC incidence in East Asia (27/100,000) [4]. Many studies have shown that the early detection of BC plays a vital role in patient survival. Further, a delay in the diagnosis and subsequent treatment can lead to worsening of morbidity and mortality [5, 6]. From the time of onset to that of diagnosis, patients may experience disease progression, which could lead to tumor growth, and consequently, worse outcomes [7]. Stages at diagnosis differ among countries with different incomes. A report showed that more than 70% of women with BC in developed countries had disease stage I or II, compared with 20% to 60% in low and middle-income countries [8]. A commercial report showed that in China, nearly two-thirds of BC patients were diagnosed with advanced disease, which was obviously higher than that in the USA [2]. It is well-known that BC screening is an effective way to detect early-stage disease; however, in contrast to developed countries, China has no such current nationwide BC screening program because of economic and demographic factors [9]. Additionally, people are not considered to consciously have the appropriate attitudes toward such BC screening programs [10]. Many studies have shown that the level of cancer

For peer review only - http://bmjopen.bmj.com/site/about/guidelines.xhtml

awareness is a significant risk factor for early detection of BC [10, 11, 12]. Therefore, it is necessary to implement interventions aimed at increasing the comprehensive knowledge and awareness of BC symptoms and screening methods.

To promote BC awareness among Chinese women and build education programs to prevent delays in diagnosis and treatment, healthcare specialists must know their current level of understanding. Thus, we performed this cross-sectional survey in China to assess the level of awareness and knowledge of breast cancer-related symptoms and risk factors, and identify awareness-related factors.

METHODS

Study design

We designed a multicenter, case-control hospital-based study to investigate the awareness of women in 21 hospitals located in 11 provinces in Northern and Eastern China. This study was conducted between April 2012 and April 2013 and was funded by the Ministry of Health of the People's Republic of China.

Study population

All participants were of the Han ethnic group. Cases and controls were matched 1:1 for age (±3 years) , diagnosis hospital (same hospital) and timing of examination (within 2 months). The inclusion criteria for BC group were as follows: (1) newly diagnosed and histologically confirmed BC and (2) women aged between 25 and 70 years old. Women who had recurrent or metastatic BC and/or complications of other malignant tumors confirmed by clinical or pathological diagnosis were excluded from this group. The members of control group were all from the regular physical examination center in cooperation hospitals. The inclusion criteria

For peer review only - http://bmjopen.bmj.com/site/about/guidelines.xhtml

For peer review only

for the control group were as follows: (1) negative physical examination results; (2) negative ultrasound scans of breast and/or mammographic screening results; and (3) no evidence or history of cancer. The exclusion criteria for the control group were as follows: a neoplastic disease at any other site, history of cancer or other major chronic diseases. We collected data strictly according to the inclusion and exclusion criteria, and then excluded subjects whose data were incomplete or lacking. Finally, a total of 1489 case-control sets were involved.

Data collection

A self-designed structured questionnaire was previously developed to record information through person-to-person interviews. The theoretical basis of this interview questionnaire was numerous published articles and the opinions of a variety of experts in breast surgery, epidemiology, statistics, nutrition, and molecular biology. Several similar questions were asked in different sections of the questionnaire to minimize recall bias. Previously, we conducted an investigation to assess the practicality and effectiveness of the survey and by which we validated the questionnaire[9]. The final interviewer-administered questionnaire was composed of the following parts: (1) demographic characteristics, physiological and reproductive factors, such as current age, age at menarche, age at menopause, menopausal status; (2) chronic diseases and family history such as breast hyperplasia, diabetes mellitus, hypertension and family history of BC; (3) lifestyle habits: smoking, alcohol intake, dietary habits, sleeping satisfaction; (4) awareness of breast cancer-related knowledge: sources of breast cancer-related information, risk factors and early signs or symptoms of BC (cumulative scores of these relevant items were counted as the related knowledge score and behavioral prevention score); (5) medical records including the visual examination, palpation and related

For peer review only - http://bmjopen.bmj.com/site/about/guidelines.xhtml

diagnostic tests results. Additionally, for BC patients, the histological and immunohistochemical diagnosis were also evaluated.

Scoring scheme

Awareness and knowledge of BC were assessed through 15 items on risk factors and early symptoms of BC included in the questionnaire (Table 2). For each item, if respondents gave a correct response ("yes"), they scored one (1) point; if a wrong response ("no" or "do not know") was given, the score for the item was zero (0). Total scores thus ranged from 0 to 15. Then, we set a score to identify the status of respondents' awareness and knowledge of BC. Respondents with scores ranging from 0 to 8 were considered to have poor awareness and knowledge, whereas those with scores ranging from 9 to 15 points were considered to have high awareness and knowledge. Behavioral prevention was scored cumulatively by five items: participation in BC screening, breast self-examination (BSE), clinical breast examination (CBE), radiographic breast examination, and breast ultrasound examination. Its scoring rules were the same as the 15-item questionnaire, and the total scores ranged from 0 to 5. The overall life satisfaction score was cumulatively based on 12 items; high scores meant low life satisfaction, and low scores indicated high life satisfaction.

Quality control

Interviewers were selected by medical professionals and medical post-graduate students. All interviewers had completed standardized training and were certified to conduct surveys independently. The questionnaires and forms were coded twice and were entered twice by different clerks. If there were inconsistent records, professionals would manually check and correct these. We also used computer software to check the logic and reasonable range of

For peer review only

For peer review only - http://bmjopen.bmj.com/site/about/guidelines.xhtml

responses throughout the questionnaire to identify contradictory responses.

Statistical analysis

EpiData3.1 was used to create the database. Statistical methods, including Student's t-test, Pearson's $\chi 2$ test, reliability analyses, exploratory factor analysis, and univariate and multivariate logistic regression analyses, were used to identify factors related to the knowledge of BC. Odds ratios (OR) with 95% confidence intervals were also calculated. All data analyses were performed using SPSS21.0.

RESULTS

In this survey, 2978 women were all included in our final analysis. The demographic characteristics are shown in Table 1. The mean age of participants was 47.38±8.8 years. A total of 1611 subjects (54.1%) were from urban areas and 1248 (41.9%) were from rural areas. More than one-third of the population (33.9%) consisted of agricultural workers. Nearly half of the women (1422, 47.8%) had other occupations; some were teachers, drivers, or provided civil service; 441 (14.8%) were workers; and 106 (3.6%) were medical staff. Regarding subject distribution based on education status, 466 subjects (15.6%) had primary school studies or less, 887 (29.8%) had completed middle school, 914 (30.7%) had completed high school, 565 (19.0%) had college degrees and 36 (1.2%) had post-graduate degrees. Most of the responders (2810, 94.4%) were married, while 33 (1.1%) were single and 135 (4.5%) were widowed or divorced. In terms of income level, 140(4.7%) earned less than 1000 Chinese Yuan Renminbi (RMB) per month, 394 (13.2%) earned 1000–1999 RMB monthly, a quarter (24.9%) of the subjects had a monthly family income of 2000–2999 RMB, 791(26.6%) of the women had monthly family income of 3000–4999 RMB per month and 821 (27.6%)

For peer review only - http://bmjopen.bmj.com/site/about/guidelines.xhtml

subjects had a monthly family income greater than 5000 RMB.

Table 1 Main characteristics of responders (N=2,978)

**Category**	**Awarenes N**		**χ2**	***p***
	**high**	**low**		
**Present history of breast cancer**				
yes	248	1241	20.585	＜0.001
no	347	1142		
**Age group（years）**				
25-	56	149		
35-	214	723		
45-	206	950	18.655	0.001
55-	108	490		
65-	11	71		
mean（SD）	47.38（8.8）			
**Location**				
urban	465	1146	189.36	＜0.001
rural	102	1146		
**Marital status**				
married	559	2251		
single	10	23	2.257	0.324
widowed/divorced	26	109		
**Monthly household income（RMB）**				

For peer review only - http://bmjopen.bmj.com/site/about/guidelines.xhtml

＜1000	7	133		
1000-1999	53	341		
2000-2999	111	631	151.925	＜0.001
3000-4999	129	662		
≥5000	281	540		
**Occupation**				
agricultural workers	63	946		
worker	94	347	281.37	＜0.001
medical staff	68	38		
others	370	1052		
**Educational status**				
primary or less	26	440		
middle	122	765		
high	216	698	186.918	＜0.001
college	201	364		
post-graduate	16	20		
**Menopause**				
yes	175	760	1.520	0.218
no	400	1534		
**History of breast hyperplasia**				
yes	200	388	91.447	＜0.001
no	383	1954		

For peer review only - http://bmjopen.bmj.com/site/about/guidelines.xhtml

**Family history of breast cancer**					
	yes	47	98	14.228	<0.001
	no	528	2178		
**Smoking**					
	yes	7	76	7.100	0.008
	no	585	2298		
**Drinking**					
	yes	106	290	12.908	<0.001
	no	488	2080		
**BMI**					
	≤23.9	290	1105		
	24.0-27.9	220	868	1.649	0.438
	≥28.0	58	271		
**WHR**					
	<0.85	288	919	17.609	<0.001
	≥0.85	219	1063		
**Present life satisfaction**					
	high	332	1492	9.308	0.002
	low	263	891		
**Behavioral prevention score**					
	low	263	891	9.308	0.002
	high	332	1492		

For peer review only

For peer review only - http://bmjopen.bmj.com/site/about/guidelines.xhtml

Responses to questions about BC among the population are summarized in Table 2. A total of 2167 subjects (72.8%) had heard about BC as a common disease affecting women, 2253 (75.7%) thought BC screening was helpful for early detection of BC, and 2386 (80.1%) believed that early detection of BC could improve survival. In terms of the knowledge of risk factors for BC, 1884 (63.3%) knew that family history of BC was a risk factor for BC; and 381 (12.8%) knew that women whose menarche age was less than 12 years are at higher risk of developing BC; 679 (22.8%) knew that no parity and late childbirth increased the risk of BC, 495 (16.6%) knew that late menopause was associated with BC, 636 (21.4%) considered high-fat diet as a risk factor, 616 (20.7%) knew that long-term drinking habits increased BC risk and 937 (31.5%) knew that long-term use of estrogen drugs was also a risk factor for BC. Regarding the awareness of early symptoms of BC, more than half (52.7%) of the subjects were aware that a breast lump could be a symptom of BC. Awareness of other symptoms was higher when compared with our previous community-based survey results (Figure 1). In summary, 595 women (20.0%) showed high BC awareness and 2383 (80.0%) poor BC awareness.

Table 2 Responses from the 15-item questionnaire for breast cancer awareness (N=2,978)

**Questions**	**N**	**%**
**Do you know breast cancer**		
Yes	2167	72.8
No	736	24.7
**Do you think screening is helpful for early detection of breast cancer**		

For peer review only - http://bmjopen.bmj.com/site/about/guidelines.xhtml

Yes	2253	75.7
No	668	22.4
**Do you think the early detection of breast cancer can improve survival**		
Yes	2386	80.1
No	536	18.0
Knowledge about breast symptom		
**Local discomfort in breast**		
Yes	1107	37.2
No	657	22.1
Don't know	1176	39.5
**Lump in breast**		
Yes	1569	52.7
No	418	14.0
Don't know	953	32.0
**Axillary nodes**		
Yes	1001	33.6
No	369	12.4
Don't know	1561	52.4
**Nipple retraction**		
Yes	790	26.5
No	367	12.3

For peer review only

Don't know	1691	56.8
**Nipple discharge liquid**		
Yes	875	29.4
No	367	12.3
Don't know	1691	56.8
Related factors of breast cancer		
**Menarche at age before 12**		
Yes	381	12.8
No	471	15.8
Don't know	2088	70.1
**No parity or late childbirth**		
Yes	679	22.8
No	377	12.7
Don't know	1880	63.1
**Menopause at a late age**		
Yes	495	16.6
No	380	12.8
Don't know	2062	69.2
**Long time drinking**		
Yes	616	20.7
No	369	12.4
Don't know	1947	65.4

For peer review only - http://bmjopen.bmj.com/site/about/guidelines.xhtml

**High-fat diets**		
Yes	636	21.4
No	353	11.9
Don't know	1947	65.4
**Long-term use of estrogen drugs**		
Yes	937	31.5
No	244	8.2
Don't know	1752	58.8
**Family history of breast cancer**		
Yes	1884	63.3
No	158	5.3
Don't know	895	30.1
**Awareness of breast cancer**		
Highly aware	595	20.0
Poorly aware	2383	80.0

Regarding the subjects' BC behavioral prevention, 1109 (37.2%) women had performed BSE at least once, 1020 of the 2978 women (37.2%) had undergone clinical breast examination, 35.9% had received breast ultrasound and 22.2% had undergone a radiographic breast examination. We set a 3-point cut-off value to distinguish high and low awareness of breast examination, and we found that more than half of the women (1824, 61.2%) had high awareness of behavioral prevention.

Most of the subjects obtained their information on BC from television broadcast and relatives

For peer review only - http://bmjopen.bmj.com/site/about/guidelines.xhtml

or friends with BC, which accounted for 30.6% and 29.6% of all women, respectively (Figure 2).

Results for univariate analysis indicated that awareness and knowledge of BC were related to history of BC, age, location, education status, occupation, household monthly income, social status, history of breast hyperplasia, family history of BC, drinking, smoking, physical training execution, waist-hip ratio, present life satisfaction, and behavioral prevention score. All significant variables were included in multivariate analysis. Multivariate analysis identified that age (OR: 0.843 95.0%CI: 0.740-0.961), occupation (agricultural worker vs. worker, medical staff: OR=3.066, 95% CI: 1.999–4.703; OR=12.831, 95% CI: 6.998–23.523, respectively), social status (OR: 0.644, 95%CI: 0.531–0.780), history of breast hyperplasia (OR: 1.684, 95%CI: 1.273–2.228), and behavioral prevention score (OR: 4.407, 95%CI: 3.433–5.657) were independently correlated with BC awareness and knowledge (Table 3) through stepwise method.

Table 3 Multivariate analysis of factors related to knowledge of breast cancer

	B	*S.E.*	*Wald*	*p*	*OR*	*95.0%CI*
Age	-0.170	0.067	6.511	0.011	0.843	0.740-0.961
Occupation						
agricultural worker				<0.001	1.000*	
worker	1.120	0.218	26.335	<0.001	3.066	1.999-4.703
medical staff	2.552	0.309	68.078	<0.001	12.831	6.998-23.523
other	1.238	0.183	45.721	<0.001	3.448	2.408-4.936
Household social status	-0.441	0.098	20.292	<0.001	0.644	0.531-0.780

For peer review only - http://bmjopen.bmj.com/site/about/guidelines.xhtml

History of breast hyperplasia	0.521	0.143	13.298	<0.001	1.684	1.273-2.228
Behavioral prevention score	1.483	0.127	135.509	<0.001	4.407	3.433-5.657

* Reference.

Occupation: "Other" includes occupations such as teacher, civil servant, individual business, driver, service, company employee, and housewife.

Household social status, reference: low social status.

History of breast hyperplasia, reference: absence of breast hyperplasia history.

Behavioral prevention score was a cumulative score of 5 items; reference: low scores.

RMB, Chinese Yuan Renminbi; BMI, body mass index; WHR, waist-hip ratio

Reliability and construct validity and internal consistency reliability estimates of the 15-item scale of awareness and knowledge of BC were calculated using Cronbach's alpha. The α-coefficient for the total scale was 0.902, which was considered acceptable for internal consistency reliability [13]. Exploratory factor analysis was conducted to explore construct validity. The Kaiser–Meyer–Olkin measure produced a coefficient of 0.883, indicative of excellent sampling adequacy. Bartlett's test of sphericity produced a value of 23825.328 ($p <$ 0.001), indicating that the correlation matrix was unlikely to be an identity matrix and was therefore suitable for factor analysis [14].

DISCUSSION

In this hospital-based study, we evaluated the level of BC awareness among Chinese women. Results showed that most participants had poor awareness regarding BC (80.0%), which is similar to our previous community-based study (81.4% of subjects had poor awareness). This seems to be a common phenomenon both in developing and developed countries, although the proportion of women with high awareness in developed countries was reportedly higher than that in developing countries or regions [15, 16].

It has also been reported that different approaches for obtaining cancer-related knowledge

For peer review only - http://bmjopen.bmj.com/site/about/guidelines.xhtml

influenced the level of awareness of BC [17]. We investigated the resources by which women obtained knowledge of BC, and we found that the majority of women obtained BC information through traditional media such as TV broadcasts (30.6%) and their friends or relatives with BC (29.6%). The Internet has developed rapidly and become widespread, but in the present study, women failed to benefit from it in terms of BC awareness (8.6%). Less women participated in special lectures about BC (2.5%), which indicated that the effort to publicize such events for BC were likely insufficient. A study comparing non-Hispanic and Hispanic college women revealed that the Internet was the most common information resource (75%) [18], while a Spanish survey indicated that the main sources of information were television, press, family, and friends [19]. In Malaysia and Cameroon, television was still the major resource for obtaining knowledge [20, 21]. It was worth noting that an increasing number of people are using the Internet to obtain information on diseases such as cancer. However, in our country, women were not accustomed to employing the Internet to search for cancer-related information, which may be a possible consequence of cultural and economic diversity.

In our study, more than two-thirds (72.8%) of the women knew or had heard about BC, but their in-depth knowledge of the early symptoms of BC and risk factors was insufficient. Although more than half (52.7%) of the women knew that the presence of a lump in the breast was a BC symptom and family history was an important risk factor for BC, the proportion of women who knew other BC symptoms and BC risk factors was low (overall, less than 40%). These results were consistent with our previous study [9] (Figure 1), which indicated that although most women in China knew or had heard about BC, their in-depth knowledge of BC

For peer review only - http://bmjopen.bmj.com/site/about/guidelines.xhtml

needed to be urgently improved. Studies from other countries showed consistent results [5, 15, 22, 23], especially those conducted in developing countries. In developed countries, the proportion of women reporting cancer-related symptoms was higher to a certain extent; this may be a possible consequence of their higher living standards, a greater consciousness of health, and more social publicity.

Many studies found a close relationship between age and awareness and knowledge of BC [10, 22, 24, 25]. The results of a study by Mandelblatt J et al. revealed that the level of knowledge of BC decreased as the age of responders increased [26]. Several articles researching Indian women also yielded similar results [10, 27]. However, a study completed by Sen et al. showed that older women were more interested in BC knowledge than younger women. Okobia et al. also arrived at the same conclusion among Nigerian women [28]. In our study, we found that age was related to BC awareness as well (OR=0.843, 95% CI: 0.740–0.961). Younger women tended to have more awareness and knowledge of BC although older women were at higher risk of BC development. We speculated that young women were more likely to focus on self-health conditions and be active learners and to access available information. From the above, it is urgent to improve the awareness and knowledge of older women to decrease the incidence of malignant breast tumors in this population.

The relationship between occupation and the level of awareness and knowledge on BC were also demonstrated in this study. Workers and medical staff tended to be more aware of the symptoms and risk factors for BC, while the awareness of agricultural workers was significantly poorer. Similar results were observed in many other studies including our community-based population survey [9, 29, 30]. Nonetheless, an Iranian study showed that the

For peer review only - http://bmjopen.bmj.com/site/about/guidelines.xhtml

awareness of BC of rural women was moderate; this may indicate the existence of ethnic and population differences [11]. As is shown in both community- and hospital-based studies, medical personnel were more aware of BC than agricultural workers (OR: 4.774, 95% CI: 4.316-5.281), likely benefiting from easier access to relevant knowledge.

Many studies revealed that education level was a major determinant of BC awareness, increased awareness was associated with higher levels of education. Terzioglu [31] stated that the awareness of BC was attributed to the respondents' education level. This was also stated in the study by Manas Kotepui [32]. The univariate analysis in our study consistently showed that awareness and knowledge of BC were related to education status; however, the result was not shown in our multivariate analysis. The study by Cannas Kwok [33] had a similar conclusion among Arabic Australia women, in whom demographic characteristics, such as education level, were a negative factor. Education level was associated with several other factors such as occupation, income, among others, but further research is warranted to clarify other factors involved.

Women with a history of breast hyperplasia tended to know more about BC in our study (OR: 1.684, 95%CI: 1.273–2.228). In some cases, the symptoms of breast hyperplasia may be similar to those of cancer; thus, women who had been diagnosed with breast hyperplasia were likely to pay more attention to breast health and to take better precautions. Breast hyperplasia may cause breast tenderness and other symptoms, but it may also serve as a forewarning for increased self-health awareness. This result was consistent with the research by Kosgeroglu et al. [34], in which it was shown that if women were diagnosed previously with benign breast diseases, they would make efforts to obtain sufficient knowledge of BC.

For peer review only - http://bmjopen.bmj.com/site/about/guidelines.xhtml

Regarding behavioral prevention of BC, our study showed that 61.2% of women adequately and consciously performed breast examination. Among the women with high awareness of BC, most had accepted breast-related examinations and 61.2% of them obtained a high behavioral prevention score. However, the proportions of women who accepted BSE, CBE, breast ultrasound or breast radiograph were still low (less than 40%). Rates of BSE practices, clinical examination, and radiographic screening were lower than those reported in the studies of Whitman et al. [35] and C Kwok, et al. [36] (55.3% and 90% had a mammogram in Chicago and Australia, respectively). The cause for this difference may be the lack of related knowledge and insufficient guidance of treatment. The relationship between early detection and BSE remains controversial [9], but most scholars still believe that self-examination probably improves awareness and might play an important role in nationwide programs for earlier-stage detection in China [37,38]. From this point of view, breast examination is helpful for early diagnosis and decreased mortality.

We compared the results with our earlier study [9]. Results showed that the proportion of women with poor awareness regarding BC was lower than that of community-based sample (80.0% vs 81.4%). As to the awareness of breast cancer awareness, we also found higher percentages in hospital-based women (Figure 1). It can be implied that medical community played an important role on it. Some studies showed the significance of medical staff on increasing level of cancer awareness. High levels of awareness about common health issues such as breast cancer was showed in Gökay Terzioğlu's study based on Turkish population, as a consequence of direct communication with professional doctors [31]. Further study on importance of medical community in China should be conducted. When compared with the

For peer review only - http://bmjopen.bmj.com/site/about/guidelines.xhtml

advanced countries, the level of BC awareness in China was still lower, which was caused by many reasons. Financial obstacle was important one of reasons [8]. In China, average household income level could not catch up with that in advanced countries in a short time. Although there are no major differences socially, culturally, and economically, the heightened awareness might be due to better infrastructure, advanced technology, and educational facilities available in the advanced regions [39, 40]. Developing countries provided women with limited access to professional knowledge and affordable quality healthcare treatment. Meantime, breast cancer screening was neither cost-effective nor feasible [41].

In this study, the result of the reliability index was 0.902 and the validity was 0.883, while the results in our previous study were 0.910 and 0.870, respectively. We used the same questionnaire in these two studies, but we still did not have uniform standards and methods for measuring breast cancer-related knowledge evaluation. Thus, we consider that a standard measurement of breast cancer-related knowledge should be developed, which will be a part of important content of our further study. Additionally, the reality and validity of our 5-items questionnaire for assessing levels of behavioral prevention was not good enough for this assessment (Cronbach's alpha 0.769 and KMO coefficient 0.780), which limited the results of the study in terms of BC practices.

CONCLUSIONS

Combined with our previous study, we concluded that most women were aware of BC as a disease entity, but their in-depth knowledge of the disease was poor. BC awareness, increased publicity, and education programs are necessary and urgent, especially for older women and agricultural workers.

For peer review only - http://bmjopen.bmj.com/site/about/guidelines.xhtml

Acknowledgments We would like to thank all of the subjects involved in the study for their participation.

Contributors: ZGY and LYL conceived and designed the interviews; LXY, FW, LL, QZ, YYL, DZG and QYF performed the interviews; YJW, YJX and LYL analyzed the data; FZ and ZBM prepared the tables and figures; LYL, YJW and FW wrote the paper; ZGY supplied suggestions**.**

Funding This research was funded by the minister-affiliated hospital key project of the Ministry of Health of the People's Republic of China (Establishment and improvement of high-risk populations screening and evaluation system for breast cancer) , the project of National Natural Science Foundation of China(NO:81602912) and the project of the Second Hospital of Shandong University (NO:S2015010014).

Competing interests The authors declare that they have no competing interests.

Ethics approval The study protocol and procedures were approved by the Institutional Review Board at the Second Hospital of Shandong University. Before conducting the interviews and under the investigators' guidance, all participants provided written informed consent.

References

For peer review only - http://bmjopen.bmj.com/site/about/guidelines.xhtml

1. DeSantis CE, Bray F, Ferlay J, Lortet-Tieulent J, Anderson BO, Jemal A. International Variation in Female Breast Cancer Incidence and Mortality Rates. Cancer Epidemiol Biomarkers Prev. 2015. 24(10): 1495-506.

2. Fan L, Strasser-Weippl K, Li JJ, et al. Breast cancer in China. Lancet Oncol. 2014. 15(7): e279-89.

3. Chen W, Zheng R, Baade PD, et al. Cancer statistics in China, 2015. CA: a cancer journal for clinicians. 2016 Mar-Apr;66(2):115-32.

4. GLOBOCAN: Estimated Cancer Incidence, Mortality and Prevalence Worldwide; 2012. http://globocan.iarc.fr/Default.aspx Accessed 17 March 2014.

5. Gupta A, Shridhar K, Dhillon PK. A review of breast cancer awareness among women in India: Cancer literate or awareness deficit. Eur J Cancer. 2015. 51(14): 2058-66.

6. Abu-Helalah AM, Alshraideh AH, Al-Hanaqtah M, Da'na M, Al-Omari A, Mubaidin R. Delay in Presentation, Diagnosis, and Treatment for Breast Cancer Patients in Jordan. Breast J. 2016. 22(2): 213-7.

7. Webber C, Jiang L, Grunfeld E, Groome PA. Identifying predictors of delayed diagnoses in symptomatic breast cancer: a scoping review. Eur J Cancer Care (Engl). 2016 .

8. Salih AM, Alfaki MM, Alam-Elhuda DM, Nouradyem MM. Factors Delaying Presentation of Sudanese Breast Cancer Patients: an Analysis Using Andersen's Model. Asian Pac J Cancer Prev. 2016. 17(4): 2105-10.

9. Liu LY, Wang F, Yu LX, et al. Breast cancer awareness among women in Eastern China: a cross-sectional study. BMC Public Health. 2014. 14: 1004.

10. Dey S, Mishra A, Govil J, Dhillon PK. Breast Cancer Awareness at the Community

For peer review only

For peer review only - http://bmjopen.bmj.com/site/about/guidelines.xhtml

Level among Women in Delhi, India. Asian Pac J Cancer Prev. 2015. 16(13): 5243-51.

11. Balouchi A, Shahdadi H, AlKhasawneh E, et al. Rural Women's Awareness about Breast Cancer in Southeastern Iran: a Cross-Sectional Study. Asian Pac J Cancer Prev. 2016. 17(4): 1875-9.

12. Cruz-Castillo AB, Hernández-Valero MA, Hovick SR, Campuzano-González ME, Karam-Calderón MA, Bustamante-Montes LP. A Study on the Knowledge, Perception, and Use of Breast Cancer Screening Methods and Quality of Care Among Women from Central Mexico. J Cancer Educ. 2015. 30(3): 453-9.

13. Health measurement scales: a practical guide to their development and use (5th edition). Aust N Z J Public Health. 2016. 40(3): 294-5.

14. Tabachnick BG, Fidell LS: Using Multivariate Statistics. New York: Allyn & Bacon; 2001.

15. Forbes LJ, Atkins L, Thurnham A, Layburn J, Haste F, Ramirez AJ. Breast cancer awareness and barriers to symptomatic presentation among women from different ethnic groups in East London. Br J Cancer. 2011. 105(10): 1474-9.

16. Oluwatosin OA, Oladepo O: Knowledge of breast cancer and its early detection measures among rural women in Akinyele Local Government Area, Ibadan, Nigeria. BMC Cancer 2006, 6:271.

17. Lee EW, Shin M, Kawaja A, Ho SS. The Augmented Cognitive Mediation Model: Examining Antecedents of Factual and Structural Breast Cancer Knowledge Among Singaporean Women. J Health Commun. 2016. 21(5): 583-92.

18. Kratzke C, Amatya A, Vilchis H. Breast cancer prevention knowledge, beliefs, and

For peer review only - http://bmjopen.bmj.com/site/about/guidelines.xhtml

information sources between non-Hispanic and Hispanic college women for risk reduction focus. J Community Health. 2015. 40(1): 124-30.

19. Baena-Cañada JM, Rosado-Varela P, Expósito-Álvarez I, González-Guerrero M, Nieto-Vera J, Benítez-Rodríguez E. Women's perceptions of breast cancer screening. Spanish screening programme survey. Breast. 2014. 23(6): 883-8.

20. Akhtari-Zavare M, Ghanbari-Baghestan A, Latiff LA, Khaniki H. Breast cancer prevention information seeking behavior and interest on cell phone and text use: a cross-sectional study in Malaysia. Asian Pac J Cancer Prev. 2015. 16(4): 1337-41.

21. Nde FP, Assob JC, Kwenti TE, Njunda AL, Tainenbe TR. Knowledge, attitude and practice of breast self-examination among female undergraduate students in the University of Buea. BMC Res Notes. 2015. 8: 43.

22. Aydogan U, Doganer YC, Kilbas Z, et al. Predictors of knowledge level and awareness towards breast cancer among Turkish females. Asian Pac J Cancer Prev. 2015. 16(1): 275-82.

23. Khan TM, Leong JP, Ming LC, Khan AH. Association of Knowledge and Cultural Perceptions of Malaysian Women with Delay in Diagnosis and Treatment of Breast Cancer: a Systematic Review. Asian Pac J Cancer Prev. 2015. 16(13): 5349-57.

24. Grunfeld EA, Ramirez AJ, Hunter MS, Richards MA. Women's knowledge and beliefs regarding breast cancer. Br J Cancer. 2002. 86(9): 1373-8.

25. Linsell L, Burgess CC, Ramirez AJ. Breast cancer awareness among older women. Br J Cancer. 2008. 99(8): 1221-5.

26. Mandelblatt J, Kaufman E, Sheppard VB, et al. Breast cancer prevention in community clinics: will low-income Latina patients participate in clinical trials. Prev Med. 2005. 40(6):

For peer review only - http://bmjopen.bmj.com/site/about/guidelines.xhtml

611-8.

27. Shalini, Varghese D, Nayak M. Awareness and impact of education on breast self examination among college going girls. Indian J Palliat Care. 2011. 17(2): 150-4.

28. Okobia MN, Bunker CH, Okonofua FE, Osime U. Knowledge, attitude and practice of Nigerian women towards breast cancer: a cross-sectional study. World J Surg Oncol. 2006. 4: 11.

29. Santorelli ML, Hirshfield KM, Steinberg MB, Rhoads GG, Lin Y, Demissie K. Hormonal therapy for breast cancer and diabetes incidence among postmenopausal women. Ann Epidemiol. 2016 .

30. Mandelblatt JS, Huang K, Makgoeng SB, et al. Preliminary Development and Evaluation of an Algorithm to Identify Breast Cancer Chemotherapy Toxicities Using Electronic Medical Records and Administrative Data. J Oncol Pract. 2014 .

31. Terzioğlu, Özgü E, Kılıç, Yıldız, Güngör T. Evaluation of Breast Cancer Knowledge and Awareness Among Hospital Staff in a Women Heath Hospital in Turkey. J Cancer Educ. 2016 .

32. Kotepui M, Piwkham D, Chupeerach C, Duangmano S. Knowledge, attitudes and practice of breast cancer screening among female personnel of Walailak University. Health Expect. 2015. 18(6): 3069-78.

33. Kwok C;Endrawes G;Lee CF Cultural Beliefs and Attitudes About Breast Cancer and Screening Practices Among Arabic Women in Australia. Cancer Nurs.2016.39 (5):367-74.

34. Kosgeroglu N, Ayranci U, Ozerdogan N, et al (2011). Knowledge of women on early diagnosis methods and risk factors for breast cancer in a province of Western Turkey: A

For peer review only - http://bmjopen.bmj.com/site/about/guidelines.xhtml

descriptive study. Pakistan J Med Sci, 27, 646-50.

35. Whitman S, Shah AM, Silva A, Ansell D. Mammography screening in six diverse communities in Chicago--a population study. Cancer Detect Prev. 2007. 31(2): 166-72.

36. Kwok C, Tranberg R, Lee FC. Breast cancer knowledge, attitudes and screening behaviors among Indian-Australian women. Eur J Oncol Nurs. 2015. 19(6): 701-6.

37. Thomas DB, Gao DL, Ray RM, et al. Randomized trial of breast self-examination in Shanghai: final results. J Natl Cancer Inst 2002, 94:1445–7.

38. Plesnicar A, Golicnik M, Fazarinc IK, et al. Attitudes of midwifery students towards teaching breast-self examination. Radiol Oncol 2010, 44:52–6.

39. Chalkidou K, Marquez P, Dhillon PK, et al. Evidence-informed frameworks for cost-effective cancer care and prevention in low, middle, and high-income countries. The Lancet. Oncology. 2014 Mar;15(3):e119-31.

40. Almutairi KM, Ahmad M, Vinluan JM, Almutairi A. Random Cross-Sectional Determination of the Level of Awareness Among Female Saudi Patients About Breast Cancer. Journal of cancer education. 2016 Mar;31(1):131-5.

41. DeSantis CE, Bray F, Ferlay J, et al. International Variation in Female Breast Cancer Incidence and Mortality Rates. Cancer epidemiology, biomarkers & prevention. 2015 Oct;24(10):1495-506.

Figure 1 Comparison of proportions of women aware of breast cancer symptoms between community-based and hospital-based studies

Figure 2 Proportions of sources of information of breast cancer for women

For peer review only

For peer review only - http://bmjopen.bmj.com/site/about/guidelines.xhtml

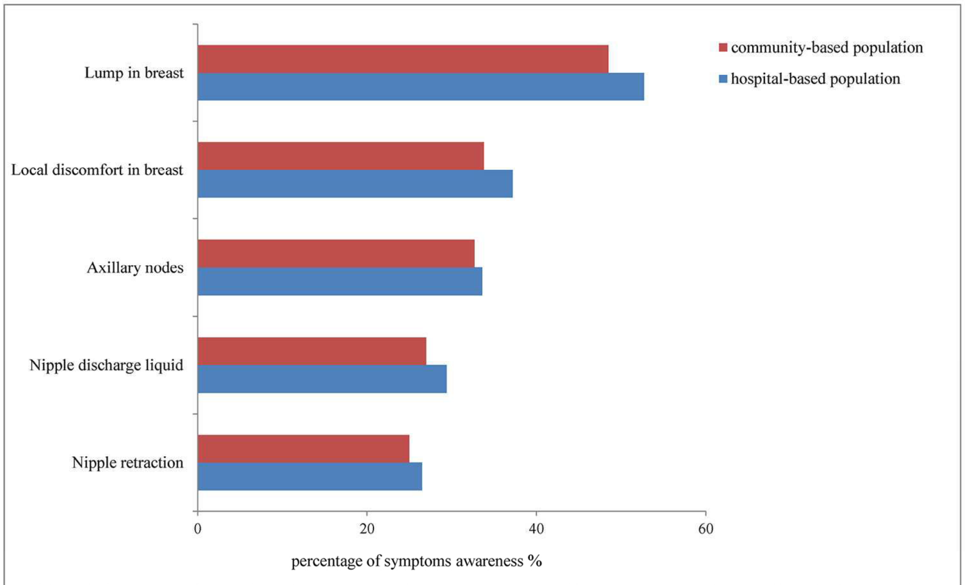

Figure 1 Comparison of proportions of women aware of breast cancer symptoms between community-based and hospital-based studies

Figure 1 Comparison of proportions of women aware of breast cancer symptoms between community-based and hospital-based studies

152x101mm (300 x 300 DPI)

For peer review only - http://bmjopen.bmj.com/site/about/guidelines.xhtml

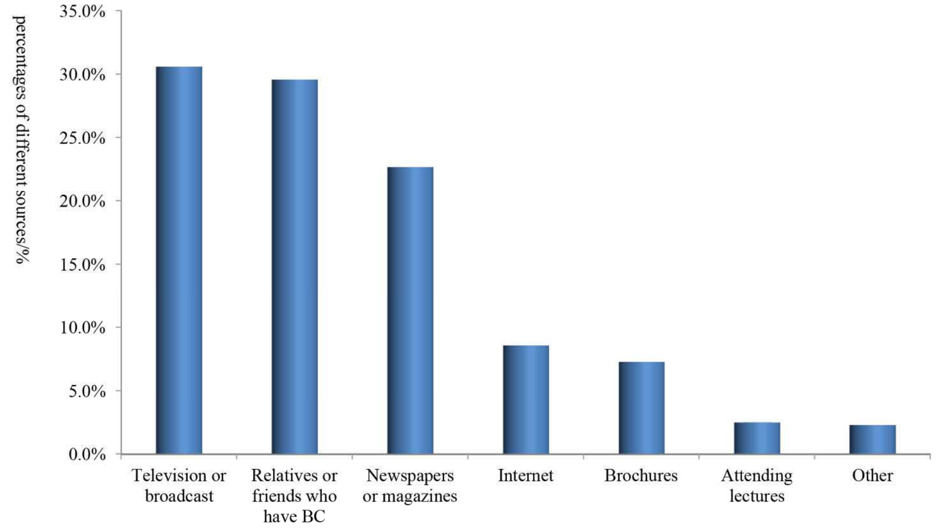

Figure 2 Proportions of sources of information of breast cancer for women

Figure 2 Proportions of sources of information of breast cancer for women

174x108mm (300 x 300 DPI)

STROBE Statement—Checklist of items that should be included in reports of ***case-control studies***

	Item No	Recommendation
**Title and abstract**	1	(*a*) Indicate the study's design with a commonly used term in the title or the abstract Page 2-3
		(*b*) Provide in the abstract an informative and balanced summary of what was done and what was found Page 2-3
**Introduction**		
Background/rationale	2	Explain the scientific background and rationale for the investigation being reported Page 4-5
Objectives	3	State specific objectives, including any prespecified hypotheses Page 5
**Methods**		
Study design	4	Present key elements of study design early in the paper Page 5
Setting	5	Describe the setting, locations, and relevant dates, including periods of recruitment, exposure, follow-up, and data collection Page 5-6
Participants	6	(*a*) Give the eligibility criteria, and the sources and methods of case ascertainment and control selection. Give the rationale for the choice of cases and controls Page 5-6
		(*b*) For matched studies, give matching criteria and the number of controls per case Page 5-6
Variables	7	Clearly define all outcomes, exposures, predictors, potential confounders, and effect modifiers. Give diagnostic criteria, if applicable Page 6-7
Data sources/ measurement	8*	For each variable of interest, give sources of data and details of methods of assessment (measurement). Describe comparability of assessment methods if there is more than one group Page 6-7
Bias	9	Describe any efforts to address potential sources of bias Page 7
Study size	10	Explain how the study size was arrived at Page 5
Quantitative variables	11	Explain how quantitative variables were handled in the analyses. If applicable, describe which groupings were chosen and why Page 6-7
Statistical methods	12	(*a*) Describe all statistical methods, including those used to control for confounding Page 8
		(*b*) Describe any methods used to examine subgroups and interactions Page 8
		(*c*) Explain how missing data were addressed Page 7
		(*d*) If applicable, explain how matching of cases and controls was addressed Page 5
		(*e*) Describe any sensitivity analyses Page 8
**Results**		
Participants	13*	(a) Report numbers of individuals at each stage of study—eg numbers potentially eligible, examined for eligibility, confirmed eligible, included in the study, completing follow-up, and analysed Page 8
		(b) Give reasons for non-participation at each stage
		(c) Consider use of a flow diagram
Descriptive data	14*	(a) Give characteristics of study participants (eg demographic, clinical, social) and information on exposures and potential confounders Page 8
		(b) Indicate number of participants with missing data for each variable of interest
Outcome data	15*	Report numbers in each exposure category, or summary measures of exposure Page8-11

For peer review only

Main results	16	(*a*) Give unadjusted estimates and, if applicable, confounder-adjusted estimates and their precision (eg, 95% confidence interval). Make clear which confounders were adjusted for and why they were included Page 8-15
		(*b*) Report category boundaries when continuous variables were categorized Page 8-15
		(*c*) If relevant, consider translating estimates of relative risk into absolute risk for a meaningful time period Page 16-17
Other analyses	17	Report other analyses done—eg analyses of subgroups and interactions, and sensitivity analyses
**Discussion**		
Key results	18	Summarise key results with reference to study objectives Page 17-21
Limitations	19	Discuss limitations of the study, taking into account sources of potential bias or imprecision. Discuss both direction and magnitude of any potential bias Page 17-21
Interpretation	20	Give a cautious overall interpretation of results considering objectives, limitations, multiplicity of analyses, results from similar studies, and other relevant evidence Page 21
Generalisability	21	Discuss the generalisability (external validity) of the study results Page 17
**Other information**		
Funding	22	Give the source of funding and the role of the funders for the present study and, if applicable, for the original study on which the present article is based Page 22

*Give information separately for cases and controls.

Note: An Explanation and Elaboration article discusses each checklist item and gives methodological background and published examples of transparent reporting. The STROBE checklist is best used in conjunction with this article (freely available on the Web sites of PLoS Medicine at http://www.plosmedicine.org/, Annals of Internal Medicine at http://www.annals.org/, and Epidemiology at http://www.epidem.com/). Information on the STROBE Initiative is available at http://www.strobe-statement.org.

For peer review only

For peer review only - http://bmjopen.bmj.com/site/about/guidelines.xhtml

Factors Associated with Insufficient Awareness of Breast Cancer among Women in Northern and Eastern China： a case-control study

Journal:	*BMJ Open*
Manuscript ID	bmjopen-2017-018523.R2
Article Type:	Research
Date Submitted by the Author:	16-Jan-2018
Complete List of Authors:	Liu, Li-Yuan; the Second Hospital of Shandong University, breast surgery; Shandong University, Institute of Translational Medicine of Breast Disease Prevention and Treatment<br>Wang, Yong-Jiu; the Second Hospital of Shandong University, breast surgery; Shandong University, Institute of Translational Medicine of Breast Disease Prevention and Treatment<br>Wang, Fei; the Second Hospital of Shandong University, breast surgery; Shandong University, Institute of Translational Medicine of Breast Disease Prevention and Treatment<br>Yu, Li-Xiang; the Second Hospital of Shandong University, breast surgery; Shandong University, Institute of Translational Medicine of Breast Disease Prevention and Treatment<br>Xiang, Yu-Juan; the Second Hospital of Shandong University, breast surgery; Shandong University, Institute of Translational Medicine of Breast Disease Prevention and Treatment<br>Zhou, Fei; the Second Hospital of Shandong University, breast surgery; Shandong University, Institute of Translational Medicine of Breast Disease Prevention and Treatment<br>Li, Liang; the Second Hospital of Shandong University, breast surgery; Shandong University, Institute of Translational Medicine of Breast Disease Prevention and Treatment<br>Zhang, Qiang; the Second Hospital of Shandong University, breast surgery; Shandong University, Institute of Translational Medicine of Breast Disease Prevention and Treatment<br>Fu, Qin-Ye; the Second Hospital of Shandong University, breast surgery; Shandong University, Institute of Translational Medicine of Breast Disease Prevention and Treatment<br>Ma, Zhong-Bing; the Second Hospital of Shandong University, breast surgery; Shandong University, Institute of Translational Medicine of Breast Disease Prevention and Treatment<br>Gao, De-Zong; the Second Hospital of Shandong University, breast surgery; Shandong University, Institute of Translational Medicine of Breast Disease Prevention and Treatment<br>Li, Yu-Yang; the Second Hospital of Shandong University, breast surgery<br>Yu, Zhi-Gang; the Second Hospital of Shandong University, breast surgery; Shandong University, Institute of Translational Medicine of Breast Disease Prevention and Treatment
**Primary Subject Heading**:	Public health

Secondary Subject Heading:	Oncology
Keywords:	Breast cancer, Awareness, Knowledge, Chinese women

SCHOLARONE™ Manuscripts

For peer review only

Factors Associated with Insufficient Awareness of Breast Cancer among Women in Northern and Eastern China： a case-control study

Li-Yuan Liu[1,2], Yong-Jiu Wang[1,2], Fei Wang[1,2], Li-Xiang Yu[1,2], Yu-Juan Xiang[1,2], Fei Zhou[1,2], Liang Li[1,2], Qiang Zhang[1,2], Qin-Ye Fu[1,2], Zhong-Bing Ma[1,2], De-Zong Gao[1,2], Yu-Yang Li[1], Zhi-Gang Yu[1,2]

[1] Department of Breast Surgery, the Second Hospital of Shandong University, Jinan, China

[2] Institute of Translational Medicine of Breast Disease Prevention and Treatment, Shandong University, Jinan, 250033, Shandong China

E-mail: Li-Yuan Liu: liuliyuanforever@163.com, Yong-Jiu Wang: wyj737@163.com, Fei Wang: wangfei4@medmail.com.cn, Li-Xiang Yu: ylx@medmail.com.cn, Yu-Juan Xiang: xxiangyujuan@126.com, Fei Zhou: zflywin@163.com, Liang Li: liliang19772004@sina.com, Qiang Zhang: vomiting@medmail.com.cn, Qin-Ye Fu: fqyhome@163.com, Zhong-Bing Ma: mazbing@163.com, De-Zong Gao: gaohill100@yeah.net, Yu-Yang Li: jinaner@tom.com, Zhi-Gang Yu*: yzg@medmail.com.cn

*Dr. Prof. Zhi-Gang Yu, MD. Department of Breast Surgery, the Second Hospital of Shandong University. Jinan 250033, Shandong, China. Tel: +8613864182636 Fax: +86-531-88960949. E-mail: yzg@medmail.com.cn

Word count: 4248

For peer review only

ABSTRACT

Objectives: To investigate the awareness and knowledge level of breast cancer among Chinese participants.

Design: Case control study

Settings: This study was based on the database of the minister-affiliated hospital key project of the Ministry of Health of the People's Republic of China that included 21 Chinese hospitals between April 2012 and April 2013.

Participants: Matched study was designed among 2978 participants with Han ethnicity aged between 25 and 70.

Primary and secondary outcome measures: Student's t-test, Pearson's $\chi 2$ test, reliability analysis, exploratory factor analysis, univariate and multivariate logistic regression analysis were performed to know the level of breast cancer knowledge and find the breast cancer awareness associated factors.

Results: 80.0% (2383/2978) of the participants had poor awareness level of breast cancer. In-depth knowledge of breast cancer such as early symptoms and risk factors was poorly found among them. TV broadcast and relatives or friends with breast cancers were the main sources of information about breast cancer. Of all participants, 72.8% (2167/2978) had heard about breast cancer as a frequent cancer affecting women, and 63.3% (1884/2978) knew that family history of breast cancer was a risk factor for breast cancer. Over half of them were aware that a breast lump could be a symptom of breast cancer. Multivariate analysis identified the following variables that predicted awareness of breast cancer: young age (OR=0.843, 95%CI: 0.740-0.961), occupation (agricultural worker) (OR = 12.831, 95% CI: 6.998–23.523),

For peer review only - http://bmjopen.bmj.com/site/about/guidelines.xhtml

For peer review only

high household social status (OR = 0.644, 95%CI: 0.531–0.780), breast hyperplasia history (OR = 1.684, 95%CI: 1.273–2.228), high behavioral prevention score (OR = 4.407, 95%CI: 3.433–5.657).

Conclusion: Most women were aware of breast cancer as a disease, but their in-depth knowledge of it was poor. More publicity and education programs to increase breast cancer awareness are necessary and urgent, especially for the aging women and agricultural workers.

Keywords: Breast cancer, Awareness, Knowledge, Chinese women

Strengths and limitations of this study

- This was a multicenter, matched case-control study designed to investigate the breast cancer awareness of women in 21 hospitals in Northern and Eastern China.
- We designed this hospital-based study to investigate the level of knowledge of breast cancer in female breast cancer patients. Meanwhile, we compared the results with our previous community-based study.
- Although we used the same questionnaire in these two studies, we still did not have uniform standards and methods for measuring knowledge evaluation. Thence a standard measurement of breast cancer-related knowledge should be developed.

For peer review only - http://bmjopen.bmj.com/site/about/guidelines.xhtml

INTRODUCTION

Breast cancer (BC) is one of the most common cancers and the leading cause of cancer-related death among women worldwide [1]. Despite that the incidence of BC was low in China, it has been increasing much faster than globally recently on account of change of diet, lifestyle and unique one-child policy [2, 3]. According to latest statistics from the National Central Cancer Registry, breast cancer is the most frequently diagnosed cancer among Chinese women in all age and ethnic groups, accounting for nearly one fifth of all cancer types. Moreover, by 2011, BC incidence had increased to 32.43/100,000, which is higher than the average BC incidence in East Asia (27/100,000) [4].

Many studies have shown that the early detection of BC plays a vital role in patient survival. Further, a delay in the diagnosis and subsequent treatment can lead to worsening of morbidity and mortality [5, 6]. From the time of onset to that of diagnosis, patients may experience disease progression, which could lead to tumor growth, and consequently, worse outcomes [7]. Stages at diagnosis differ among countries with different incomes. Salih AM et al. reported that more than 70% of women with BC in developed countries had disease stage I or II, compared with 20% to 60% in low and middle-income countries [8]. A commercial report showed that nearly two-thirds of BC patients were diagnosed with advanced disease in China, which was obviously higher than that in the USA [2]. It is well-known that BC screening is an effective way to detect early-stage disease. However, the current nationwide BC screening program is not available in China because of economic and demographic factors [9]. Additionally, people are not considered to consciously have the appropriate attitudes toward such BC screening programs [10]. Many studies have shown that the level of cancer awareness

For peer review only - http://bmjopen.bmj.com/site/about/guidelines.xhtml

is significantly related with early detection of BC [10, 11, 12]. Therefore, it is necessary to implement interventions aimed at increasing the comprehensive knowledge and awareness of BC symptoms and screening methods.

To promote BC awareness among Chinese women and build education programs to prevent delays in diagnosis and treatment, healthcare specialists must know their current level of understanding. Thus, we performed this cross-sectional survey in China to assess the level of awareness and knowledge of breast cancer-related symptoms and risk factors, and identify awareness-related factors.

METHODS

Study design

We designed a multicenter, case-control hospital-based study to investigate the awareness of women in 21 hospitals located in 11 provinces in Northern and Eastern China. This study was conducted between April 2012 and April 2013 and was funded by the Ministry of Health of the People's Republic of China.

Study population

All participants were of the Han ethnic group. Cases and controls were matched 1:1 for age (±3 years), diagnosis hospital (same hospital) and timing of examination (within 2 months). The inclusion criteria for BC group were as follows: (1) newly diagnosed and histologically confirmed BC and (2) women aged between 25 and 70 years old. Women who had recurrent or metastatic BC and/or complications of other malignant tumors confirmed by clinical or pathological diagnosis were excluded from this group. The members of control group were all from the regular physical examination center in cooperation hospitals. The inclusion criteria

For peer review only - http://bmjopen.bmj.com/site/about/guidelines.xhtml

for the control group were as follows: (1) negative physical examination results; (2) negative ultrasound scans of breast and/or mammographic screening results; and (3) no evidence or history of cancer. The exclusion criteria for the control group were as follows: a neoplastic disease at any other site, history of cancer or other major chronic diseases. We collected data strictly according to the inclusion and exclusion criteria, and then excluded subjects whose data were incomplete or lacking. Finally, a total of 1489 case-control sets were involved.

Data collection

A self-designed structured questionnaire was previously developed to record information through person-to-person interviews. The theoretical basis of this interview questionnaire was numerous published articles and the opinions of a variety of experts in breast surgery, epidemiology, statistics, nutrition, and molecular biology. Several similar questions were asked in different sections of the questionnaire to minimize recall bias. Previously, we conducted an investigation to assess the practicality and effectiveness of the survey and by which we validated the questionnaire [9]. The final interviewer-administered questionnaire was composed of the following parts: (1) demographic characteristics, physiological and reproductive factors, such as current age, age at menarche, age at menopause, menopausal status; (2) chronic diseases and family history such as breast hyperplasia, diabetes mellitus, hypertension and family history of BC; (3) lifestyle habits: smoking, alcohol intake, dietary habits, sleeping satisfaction; (4) awareness of breast cancer-related knowledge: sources of breast cancer-related information, risk factors and early signs or symptoms of BC (cumulative scores of these relevant items were counted as the related knowledge score and behavioral prevention score); (5) medical records including the visual examination, palpation and related

For peer review only - http://bmjopen.bmj.com/site/about/guidelines.xhtml

diagnostic tests results. Additionally, for BC patients, the histological and immunohistochemical diagnosis were also evaluated.

Scoring scheme

Awareness and knowledge of BC were assessed through 15 items on risk factors and early symptoms of BC included in the questionnaire (Table 1). For each item, if respondents gave a correct response ("yes"), they scored one (1) point; if a wrong response ("no" or "do not know") was given, the score for the item was zero (0). Total scores thus ranged from 0 to 15. Then, we set a score to identify the status of respondents' awareness and knowledge of BC. Respondents with scores ranging from 0 to 8 were considered to have poor awareness and knowledge, whereas those with scores ranging from 9 to 15 points were considered to have high awareness and knowledge. Behavioral prevention was scored cumulatively by five items: participation in BC screening, breast self-examination (BSE), clinical breast examination (CBE), radiographic breast examination, and breast ultrasound examination. Its scoring rules were the same as the 15-item questionnaire, and the total scores ranged from 0 to 5. The overall life satisfaction score was cumulatively based on 12 items; high scores meant low life satisfaction, and low scores indicated high life satisfaction.

Quality control

Interviewers were selected by medical professionals and medical post-graduate students. All interviewers had completed standardized training and were certified to conduct surveys independently. The questionnaires and forms were coded twice and were entered twice by different clerks. If there were inconsistent records, professionals would manually check and correct these. We also used computer software to check the logic and reasonable range of

For peer review only

For peer review only - http://bmjopen.bmj.com/site/about/guidelines.xhtml

responses throughout the questionnaire to identify contradictory responses.

Statistical analysis

EpiData3.1 was used to create the database. Statistical methods, including Student's t-test, Pearson's $\chi 2$ test, reliability analyses, exploratory factor analysis, and univariate and multivariate logistic regression analyses, were used to identify factors related to the knowledge of BC. Odds ratios (OR) with 95% confidence intervals were also calculated. All data analyses were performed using SPSS21.0.

RESULTS

In this survey, 2978 women were included in our final analysis. The demographic characteristics are shown in Table 2. The mean age of participants was 47.38±8.8 years. A total of 1611 subjects (54.1%) were from urban areas and 1248 (41.9%) were from rural areas. More than one-third of the population (33.9%) consisted of agricultural workers. Nearly half of the women (1422, 47.8%) had other occupations; some were teachers, drivers, or provided civil service; 441 (14.8%) of them were workers; and 106 (3.6%) were medical staff. Regarding subject distribution based on education status, 466 subjects (15.6%) had primary school studies or less, 887 (29.8%) had completed middle school, 914 (30.7%) had completed high school, 565 (19.0%) had college degrees and 36 (1.2%) had post-graduate degrees. Most of the responders (2810, 94.4%) were married, while 33 (1.1%) were single and 135 (4.5%) were widowed or divorced. In terms of income level, 140(4.7%) earned less than 1000 Chinese Yuan Renminbi (RMB) per month, 394 (13.2%) earned 1000–1999 RMB monthly, a quarter (24.9%) of the subjects had a monthly family income of 2000–2999 RMB, 791(26.6%) of the women had monthly family income of 3000–4999 RMB per month and 821 (27.6%)

For peer review only - http://bmjopen.bmj.com/site/about/guidelines.xhtml

subjects had a monthly family income more than 5000 RMB.

Table 1 Responses from the 15-item questionnaire for breast cancer awareness (N=2,978)

Questions	N	%
**Do you know breast cancer**		
Yes	2167	72.8
No	736	24.7
**Do you think screening is helpful for early detection of breast cancer**		
Yes	2253	75.7
No	668	22.4
**Do you think the early detection of breast cancer can improve survival**		
Yes	2386	80.1
No	536	18.0
Knowledge about breast symptom		
**Local discomfort in breast**		
Yes	1107	37.2
No	657	22.1
Don't know	1176	39.5
**Lump in breast**		
Yes	1569	52.7
No	418	14.0
Don't know	953	32.0

For peer review only - http://bmjopen.bmj.com/site/about/guidelines.xhtml

**Axillary nodes**		
Yes	1001	33.6
No	369	12.4
Don't know	1561	52.4
**Nipple retraction**		
Yes	790	26.5
No	367	12.3
Don't know	1691	56.8
**Nipple discharge liquid**		
Yes	875	29.4
No	367	12.3
Don't know	1691	56.8
Related factors of breast cancer		
**Menarche at age before 12**		
Yes	381	12.8
No	471	15.8
Don't know	2088	70.1
**No parity or late childbirth**		
Yes	679	22.8
No	377	12.7
Don't know	1880	63.1
**Menopause at a late age**		

For peer review only - http://bmjopen.bmj.com/site/about/guidelines.xhtml

Yes	495	16.6
No	380	12.8
Don't know	2062	69.2
**Long time drinking**		
Yes	616	20.7
No	369	12.4
Don't know	1947	65.4
**High-fat diets**		
Yes	636	21.4
No	353	11.9
Don't know	1947	65.4
**Long-term use of estrogen drugs**		
Yes	937	31.5
No	244	8.2
Don't know	1752	58.8
**Family history of breast cancer**		
Yes	1884	63.3
No	158	5.3
Don't know	895	30.1
**Awareness of breast cancer**		
Highly aware	595	20.0
Poorly aware	2383	80.0

For peer review only - http://bmjopen.bmj.com/site/about/guidelines.xhtml

Responses to questions about BC among the population are summarized in Table 1. A total of 2167 subjects (72.8%) had heard about BC as a common disease affecting women, 2253 (75.7%) thought BC screening was helpful for early detection of BC, and 2386 (80.1%) believed that early detection of BC could improve survival. In terms of the knowledge of risk factors for BC, 1884 (63.3%) knew that family history of BC was a risk factor for BC; and 381 (12.8%) knew that women whose menarche age was less than 12 years are at higher risk of developing BC; 679 (22.8%) knew that no parity and late childbirth increased the risk of BC, 495 (16.6%) knew that late menopause was associated with BC, 636 (21.4%) considered high-fat diet as a risk factor, 616 (20.7%) knew that long-term drinking habits increased BC risk and 937 (31.5%) knew that long-term use of estrogen drugs was also a risk factor for BC. Regarding the awareness of early symptoms of BC, more than half (52.7%) of the subjects were aware that a breast lump could be a symptom of BC. Awareness of other symptoms was higher when compared with our previous community-based survey results (Figure 1). In summary, 595 women (20.0%) showed high BC awareness and 2383 (80.0%) poor BC awareness.

Table 2 Main characteristics of responders (N=2,978)

**Category**		**Awarenes N**		**χ2**	***p***
		**high**	**low**		
**Present history of breast cancer**					
	yes	248	1241	20.585	＜0.001
	no	347	1142		
**Age group（years）**					

For peer review only - http://bmjopen.bmj.com/site/about/guidelines.xhtml

25-	56	149		
35-	214	723		
45-	206	950	18.655	0.001
55-	108	490		
65-	11	71		
mean（SD）	47.38（8.8）			
**Location**				
urban	465	1146	189.36	＜0.001
rural	102	1146		
**Marital status**				
married	559	2251		
single	10	23	2.257	0.324
widowed/divorced	26	109		
**Monthly household income（RMB）**				
＜1000	7	133		
1000-1999	53	341		
2000-2999	111	631	151.925	＜0.001
3000-4999	129	662		
≥5000	281	540		
**Occupation**				
agricultural workers	63	946	281.37	＜0.001
worker	94	347		

For peer review only - http://bmjopen.bmj.com/site/about/guidelines.xhtml

medical staff	68	38		
others	370	1052		
**Educational status**				
primary or less	26	440		
middle	122	765		
high	216	698	186.918	＜0.001
college	201	364		
post-graduate	16	20		
**Menopause**				
yes	175	760	1.520	0.218
no	400	1534		
**History of breast hyperplasia**				
yes	200	388	91.447	＜0.001
no	383	1954		
**Family history of breast cancer**				
yes	47	98	14.228	＜0.001
no	528	2178		
**Smoking**				
yes	7	76	7.100	0.008
no	585	2298		
**Drinking**				
yes	106	290	12.908	＜0.001

For peer review only - http://bmjopen.bmj.com/site/about/guidelines.xhtml

no	488	2080		
**BMI**				
≤23.9	290	1105		
24.0-27.9	220	868	1.649	0.438
≥28.0	58	271		
**WHR**				
<0.85	288	919	17.609	<0.001
≥0.85	219	1063		
**Present life satisfaction**				
high	332	1492	9.308	0.002
low	263	891		
**Behavioral prevention score**				
low	263	891	9.308	0.002
high	332	1492		

Regarding the subjects' BC behavioral prevention, 1109 (37.2%) women had performed BSE at least once, 1020 of the 2978 women (37.2%) had undergone clinical breast examination, 35.9% had received breast ultrasound and 22.2% had undergone a radiographic breast examination. We set a 3-point cut-off value to distinguish high and low awareness of breast examination, and we found that more than half of the women (1824, 61.2%) had high awareness of behavioral prevention.

Most of the subjects obtained their information on BC from television broadcast and relatives or friends with BC, which accounted for 30.6% and 29.6% of all women, respectively (Figure

For peer review only

2).

Results for univariate analysis indicated that awareness and knowledge of BC were related to history of BC, age, location, education status, occupation, household monthly income, social status, history of breast hyperplasia, family history of BC, drinking, smoking, physical training execution, waist-hip ratio, present life satisfaction, and behavioral prevention score. All significant variables were included in multivariate analysis. Multivariate analysis identified that age (OR: 0.843 95.0%CI: 0.740-0.961), occupation (agricultural worker vs. worker, medical staff: OR=3.066, 95% CI: 1.999–4.703; OR=12.831, 95% CI: 6.998–23.523, respectively), social status (OR: 0.644, 95%CI: 0.531–0.780), history of breast hyperplasia (OR: 1.684, 95%CI: 1.273–2.228), and behavioral prevention score (OR: 4.407, 95%CI: 3.433–5.657) were independently correlated with BC awareness and knowledge (Table 3) through stepwise method.

Table 3 Multivariate analysis of factors related to knowledge of breast cancer

	B	*S.E.*	*Wald*	*p*	*OR*	*95.0%CI*
Age	-0.170	0.067	6.511	0.011	0.843	0.740-0.961
Occupation						
agricultural worker				＜0.001	1.000*	
worker	1.120	0.218	26.335	＜0.001	3.066	1.999-4.703
medical staff	2.552	0.309	68.078	＜0.001	12.831	6.998-23.523
other	1.238	0.183	45.721	＜0.001	3.448	2.408-4.936
Household social status	-0.441	0.098	20.292	＜0.001	0.644	0.531-0.780
History of breast	0.521	0.143	13.298	＜0.001	1.684	1.273-2.228

For peer review only - http://bmjopen.bmj.com/site/about/guidelines.xhtml

hyperplasia						
Behavioral prevention score	1.483	0.127	135.509	<0.001	4.407	3.433-5.657

* Reference.

Occupation: "Other" includes occupations such as teacher, civil servant, individual business, driver, service, company employee, and housewife.

Household social status, reference: low social status.

History of breast hyperplasia, reference: absence of breast hyperplasia history.

Behavioral prevention score was a cumulative score of 5 items; reference: low scores.

RMB, Chinese Yuan Renminbi; BMI, body mass index; WHR, waist-hip ratio

Reliability and construct validity and internal consistency reliability estimates of the 15-item scale of awareness and knowledge of BC were calculated using Cronbach's alpha. The α-coefficient for the total scale was 0.902, which was considered acceptable for internal consistency reliability [13]. Exploratory factor analysis was conducted to explore construct validity. The Kaiser–Meyer–Olkin measure produced a coefficient of 0.883, indicative of excellent sampling adequacy. Bartlett's test of sphericity produced a value of 23825.328 ($p < 0.001$), indicating that the correlation matrix was unlikely to be an identity matrix and was therefore suitable for factor analysis [14].

DISCUSSION

In this hospital-based study, we evaluated the level of BC awareness among Chinese women. Results showed that most participants had poor awareness regarding BC (80.0%), which is similar to our previous community-based study (81.4% of subjects had poor awareness). This seems to be a common phenomenon both in developing and developed countries, although the proportion of women with high awareness in developed countries was reportedly higher than that in developing countries or regions [15, 16].

It has also been published that different approaches for obtaining cancer-related knowledge influenced the level of awareness of BC [17]. We investigated the resources by which women

For peer review only - http://bmjopen.bmj.com/site/about/guidelines.xhtml

obtained knowledge of BC, and we found that the majority of women obtained BC information through traditional media such as TV broadcasts (30.6%) and their friends or relatives with BC (29.6%). The Internet has developed rapidly and become widespread, but in the present study, women failed to benefit from it in terms of BC awareness (8.6%). Less women participated in special lectures about BC (2.5%), which indicated that the effort to publicize such events for BC were likely insufficient. A study comparing non-Hispanic and Hispanic college women revealed that the Internet was the most common information resource (75%) [18], while a Spanish survey indicated that the main sources of information were television, press, family, and friends [19]. In Malaysia and Cameroon, television was still the major resource for obtaining knowledge [20, 21]. It is worth noting that an increasing number of people are using the Internet to obtain information on diseases such as cancer. However, in our country, people were not accustomed to employing the Internet to search for cancer-related information, which may be a possible consequence of cultural and economic diversity.

In our study, more than two-thirds (72.8%) of the participants knew or had heard about BC, but their in-depth knowledge of the early symptoms of BC and risk factors was insufficient. Although more than half (52.7%) of them knew that the presence of a lump in the breast was a BC symptom and family history was an important risk factor for BC, the proportion of women who knew other BC symptoms and BC risk factors was low (overall, less than 40%). These results were consistent with our previous study [9] (Figure 1), which indicated that although most women in China knew or had heard about BC, their in-depth knowledge of BC needed to be urgently improved. Studies from other countries showed consistent results [5, 15, 22,

For peer review only - http://bmjopen.bmj.com/site/about/guidelines.xhtml

[23], especially those conducted in developing countries. In developed countries, the proportion of women reporting cancer-related symptoms was higher to a certain extent; this may be a possible consequence of their higher living standards, a greater consciousness of health, and more social publicity.

Many studies found a close relationship between age and awareness and knowledge of BC [10, 22, 24, 25]. The results of a study by Mandelblatt J et al. revealed that the level of knowledge of BC decreased as the age of responders increased [26]. Several articles researching Indian women also yielded similar results [10, 27]. However, a study completed by Sen et al. showed that older women were more interested in BC knowledge than younger women. Okobia et al. also arrived at the same conclusion among Nigerian women [28]. In our study, we found that age was related to BC awareness as well (OR=0.843, 95% CI: 0.740–0.961). Younger women tended to have more awareness and knowledge of BC although older women were at higher risk of BC development. We speculated that young women were more likely to focus on self-health conditions and be active learners and to access available information. From the above, it is urgent to improve the awareness and knowledge of older women to decrease the incidence of malignant breast tumors in this population.

The relationship between occupation and the level of awareness and knowledge on BC were also demonstrated in this study. Workers and medical staff tended to be more aware of the symptoms and risk factors for BC, while the awareness of agricultural workers was significantly poorer. Similar results were observed in many other studies including our community-based population survey [9, 29, 30]. Nonetheless, an Iranian study showed that the awareness of BC of rural women was moderate; this may indicate the existence of ethnic and

For peer review only - http://bmjopen.bmj.com/site/about/guidelines.xhtml

population differences [11]. As is shown in both community- and hospital-based studies, medical personnel were more aware of BC than agricultural workers (OR: 4.774, 95% CI: 4.316-5.281), likely benefiting from easier access to relevant knowledge.

Many studies demonstrated that education level was a major determinant of BC awareness and increased awareness was associated with higher levels of education. Terzioglu et al. [31] reported that the awareness of BC was attributed to the respondents' education level. This was also published in the study by Manas Kotepui [32]. The univariate analysis in our study consistently showed that awareness and knowledge of BC were related to education status; however, this result was not shown in our multivariate analysis. A study published in 2016 [33] had a similar conclusion among Arabic Australia women, in which demographic characteristics, such as education level, were demonstrated as a negative factor. Education level was associated with several other factors such as occupation, income, among others, but further research is warranted to clarify other factors involved.

Women with a history of breast hyperplasia tended to know more about BC in our study (OR: 1.684, 95%CI: 1.273–2.228). In some cases, the symptoms of breast hyperplasia may be similar to those of cancer; thus, women who had been diagnosed with breast hyperplasia were likely to pay more attention to breast health and to take better precautions. Breast hyperplasia may cause breast tenderness and other symptoms, but it may also serve as a forewarning for increased self-health awareness. This result was consistent with the research by Kosgeroglu et al. [34], in which it was shown that if women were diagnosed previously with benign breast diseases, they would make efforts to obtain sufficient knowledge of BC.

Regarding behavioral prevention of BC, our study showed that 61.2% of women adequately

For peer review only - http://bmjopen.bmj.com/site/about/guidelines.xhtml

and consciously performed breast examination. Among the women with high awareness of BC, most had accepted breast-related examinations and 61.2% of them obtained a high behavioral prevention score. However, the proportions of women who accepted BSE, CBE, breast ultrasound or breast radiograph were still low (less than 40%). Rates of BSE practices, clinical examination, and radiographic screening were lower than those reported in the studies of Whitman et al. [35] and C Kwok, et al. [36] (55.3% and 90% had a mammogram in Chicago and Australia, respectively). The cause for this difference may be the lack of related knowledge and insufficient guidance of treatment. The relationship between early detection and BSE remains controversial [9], but most scholars still believe that self-examination probably improves awareness and might play an important role in nationwide programs for earlier-stage detection in China [37,38]. From this point of view, breast examination is helpful for early diagnosis and decreased mortality.

We compared the results with our earlier study [9]. Results showed that the proportion of women with poor awareness of BC was lower than that of community-based sample (80.0% vs 81.4%). As to the awareness of breast cancer awareness, we also found higher percentages in hospital-based women (Figure 1). It can be implied that medical community played an important role on it. Some studies showed the significance of medical staff on increasing level of cancer awareness. High levels of awareness about common health issues such as breast cancer was shown in Gökay Terzioğlu's study based on Turkish population, as a consequence of direct communication with professional doctors [31]. Further study on importance of medical community in China should be conducted. When compared with the advanced countries, the level of BC awareness in China was lower, which was caused by many

For peer review only - http://bmjopen.bmj.com/site/about/guidelines.xhtml

reasons. Financial obstacle was important one of reasons [8]. In China, average household income level could not catch up with that in advanced countries in a short time. Although there are no major differences socially, culturally, and economically, the heightened awareness might be due to better infrastructure, advanced technology, and educational facilities available in the advanced regions [39, 40]. Developing countries provided women with limited access to professional knowledge and affordable quality healthcare treatment. Meantime, breast cancer screening was neither cost-effective nor feasible [41].

In this study, the result of the reliability index was 0.902 and the validity was 0.883, while the results in our previous study were 0.910 and 0.870, respectively. We used the same questionnaire in these two studies, but we still did not have uniform standards and methods for measuring breast cancer-related knowledge evaluation. Thus, we consider that a standard measurement of breast cancer-related knowledge should be developed, which will be a part of important content of our further study. Additionally, the reality and validity of our 5-items questionnaire for assessing levels of behavioral prevention was not good enough for this assessment (Cronbach's alpha 0.769 and KMO coefficient 0.780), which limited the results of the study in terms of BC practices.

CONCLUSIONS

Combined with our previous study, we concluded that most women were aware of BC as a disease entity, but their in-depth knowledge of the disease was poor. BC awareness, increased publicity, and education programs are necessary and urgent, especially for older women and agricultural workers.

Acknowledgments We would like to thank all of the subjects involved in the study for their

For peer review only - http://bmjopen.bmj.com/site/about/guidelines.xhtml

participation.

Contributors: ZGY and LYL conceived and designed the interviews; LXY, FW, LL, QZ, YYL, DZG and QYF performed the interviews; YJW, YJX and LYL analyzed the data; FZ and ZBM prepared the tables and figures; LYL, YJW and FW wrote the paper; ZGY supplied suggestions**.**

Funding This research was funded by the minister-affiliated hospital key project of the Ministry of Health of the People's Republic of China (Establishment and improvement of high-risk populations screening and evaluation system for breast cancer) , the project of National Natural Science Foundation of China(NO:81602912) and the project of the Second Hospital of Shandong University (NO:S2015010014).

Competing interests The authors declare that they have no competing interests.

Ethics approval The study protocol and procedures were approved by the Institutional Review Board at the Second Hospital of Shandong University. Before conducting the interviews and under the investigators' guidance, all participants provided written informed consent.

Data sharing statement Anonymous datasets are available from the corresponding author （yzg@medmail.com.cn）.

For peer review only

References

1. DeSantis CE, Bray F, Ferlay J, Lortet-Tieulent J, Anderson BO, Jemal A. International Variation in Female Breast Cancer Incidence and Mortality Rates. Cancer Epidemiol Biomarkers Prev. 2015. 24(10): 1495-506.

2. Fan L, Strasser-Weippl K, Li JJ, et al. Breast cancer in China. Lancet Oncol. 2014. 15(7): e279-89.

3. Chen W, Zheng R, Baade PD, et al. Cancer statistics in China, 2015. CA: a cancer journal for clinicians. 2016 Mar-Apr;66(2):115-32.

4. GLOBOCAN: Estimated Cancer Incidence, Mortality and Prevalence Worldwide; 2012. http://globocan.iarc.fr/Default.aspx Accessed 17 March 2014.

5. Gupta A, Shridhar K, Dhillon PK. A review of breast cancer awareness among women in India: Cancer literate or awareness deficit. Eur J Cancer. 2015. 51(14): 2058-66.

6. Abu-Helalah AM, Alshraideh AH, Al-Hanaqtah M, Da'na M, Al-Omari A, Mubaidin R. Delay in Presentation, Diagnosis, and Treatment for Breast Cancer Patients in Jordan. Breast J. 2016. 22(2): 213-7.

7. Webber C, Jiang L, Grunfeld E, Groome PA. Identifying predictors of delayed diagnoses in symptomatic breast cancer: a scoping review. Eur J Cancer Care (Engl). 2016 .

8. Salih AM, Alfaki MM, Alam-Elhuda DM, Nouradyem MM. Factors Delaying Presentation of Sudanese Breast Cancer Patients: an Analysis Using Andersen's Model. Asian Pac J Cancer Prev. 2016. 17(4): 2105-10.

9. Liu LY, Wang F, Yu LX, et al. Breast cancer awareness among women in Eastern China: a cross-sectional study. BMC Public Health. 2014. 14: 1004.

For peer review only - http://bmjopen.bmj.com/site/about/guidelines.xhtml

10. Dey S, Mishra A, Govil J, Dhillon PK. Breast Cancer Awareness at the Community Level among Women in Delhi, India. Asian Pac J Cancer Prev. 2015. 16(13): 5243-51.

11. Balouchi A, Shahdadi H, AlKhasawneh E, et al. Rural Women's Awareness about Breast Cancer in Southeastern Iran: a Cross-Sectional Study. Asian Pac J Cancer Prev. 2016. 17(4): 1875-9.

12. Cruz-Castillo AB, Hernández-Valero MA, Hovick SR, Campuzano-González ME, Karam-Calderón MA, Bustamante-Montes LP. A Study on the Knowledge, Perception, and Use of Breast Cancer Screening Methods and Quality of Care Among Women from Central Mexico. J Cancer Educ. 2015. 30(3): 453-9.

13. Health measurement scales: a practical guide to their development and use (5th edition). Aust N Z J Public Health. 2016. 40(3): 294-5.

14. Tabachnick BG, Fidell LS: Using Multivariate Statistics. New York: Allyn & Bacon; 2001.

15. Forbes LJ, Atkins L, Thurnham A, Layburn J, Haste F, Ramirez AJ. Breast cancer awareness and barriers to symptomatic presentation among women from different ethnic groups in East London. Br J Cancer. 2011. 105(10): 1474-9.

16. Oluwatosin OA, Oladepo O: Knowledge of breast cancer and its early detection measures among rural women in Akinyele Local Government Area, Ibadan, Nigeria. BMC Cancer 2006, 6:271.

17. Lee EW, Shin M, Kawaja A, Ho SS. The Augmented Cognitive Mediation Model: Examining Antecedents of Factual and Structural Breast Cancer Knowledge Among Singaporean Women. J Health Commun. 2016. 21(5): 583-92.

For peer review only - http://bmjopen.bmj.com/site/about/guidelines.xhtml

18. Kratzke C, Amatya A, Vilchis H. Breast cancer prevention knowledge, beliefs, and information sources between non-Hispanic and Hispanic college women for risk reduction focus. J Community Health. 2015. 40(1): 124-30.

19. Baena-Cañada JM, Rosado-Varela P, Expósito-Álvarez I, González-Guerrero M, Nieto-Vera J, Benítez-Rodríguez E. Women's perceptions of breast cancer screening. Spanish screening programme survey. Breast. 2014. 23(6): 883-8.

20. Akhtari-Zavare M, Ghanbari-Baghestan A, Latiff LA, Khaniki H. Breast cancer prevention information seeking behavior and interest on cell phone and text use: a cross-sectional study in Malaysia. Asian Pac J Cancer Prev. 2015. 16(4): 1337-41.

21. Nde FP, Assob JC, Kwenti TE, Njunda AL, Tainenbe TR. Knowledge, attitude and practice of breast self-examination among female undergraduate students in the University of Buea. BMC Res Notes. 2015. 8: 43.

22. Aydogan U, Doganer YC, Kilbas Z, et al. Predictors of knowledge level and awareness towards breast cancer among Turkish females. Asian Pac J Cancer Prev. 2015. 16(1): 275-82.

23. Khan TM, Leong JP, Ming LC, Khan AH. Association of Knowledge and Cultural Perceptions of Malaysian Women with Delay in Diagnosis and Treatment of Breast Cancer: a Systematic Review. Asian Pac J Cancer Prev. 2015. 16(13): 5349-57.

24. Grunfeld EA, Ramirez AJ, Hunter MS, Richards MA. Women's knowledge and beliefs regarding breast cancer. Br J Cancer. 2002. 86(9): 1373-8.

25. Linsell L, Burgess CC, Ramirez AJ. Breast cancer awareness among older women. Br J Cancer. 2008. 99(8): 1221-5.

26. Mandelblatt J, Kaufman E, Sheppard VB, et al. Breast cancer prevention in community

For peer review only - http://bmjopen.bmj.com/site/about/guidelines.xhtml

clinics: will low-income Latina patients participate in clinical trials. Prev Med. 2005. 40(6): 611-8.

27. Shalini, Varghese D, Nayak M. Awareness and impact of education on breast self examination among college going girls. Indian J Palliat Care. 2011. 17(2): 150-4.

28. Okobia MN, Bunker CH, Okonofua FE, Osime U. Knowledge, attitude and practice of Nigerian women towards breast cancer: a cross-sectional study. World J Surg Oncol. 2006. 4: 11.

29. Santorelli ML, Hirshfield KM, Steinberg MB, Rhoads GG, Lin Y, Demissie K. Hormonal therapy for breast cancer and diabetes incidence among postmenopausal women. Ann Epidemiol. 2016 .

30. Mandelblatt JS, Huang K, Makgoeng SB, et al. Preliminary Development and Evaluation of an Algorithm to Identify Breast Cancer Chemotherapy Toxicities Using Electronic Medical Records and Administrative Data. J Oncol Pract. 2014 .

31. Terzioğlu, Özgü E, Kılıç, Yıldız, Güngör T. Evaluation of Breast Cancer Knowledge and Awareness Among Hospital Staff in a Women Heath Hospital in Turkey. J Cancer Educ. 2016 .

32. Kotepui M, Piwkham D, Chupeerach C, Duangmano S. Knowledge, attitudes and practice of breast cancer screening among female personnel of Walailak University. Health Expect. 2015. 18(6): 3069-78.

33. Kwok C;Endrawes G;Lee CF Cultural Beliefs and Attitudes About Breast Cancer and Screening Practices Among Arabic Women in Australia. Cancer Nurs.2016.39 (5):367-74.

34. Kosgeroglu N, Ayranci U, Ozerdogan N, et al (2011). Knowledge of women on early

For peer review only

For peer review only - http://bmjopen.bmj.com/site/about/guidelines.xhtml

diagnosis methods and risk factors for breast cancer in a province of Western Turkey: A descriptive study. Pakistan J Med Sci, 27, 646-50.

35. Whitman S, Shah AM, Silva A, Ansell D. Mammography screening in six diverse communities in Chicago--a population study. Cancer Detect Prev. 2007. 31(2): 166-72.

36. Kwok C, Tranberg R, Lee FC. Breast cancer knowledge, attitudes and screening behaviors among Indian-Australian women. Eur J Oncol Nurs. 2015. 19(6): 701-6.

37. Thomas DB, Gao DL, Ray RM, et al. Randomized trial of breast self-examination in Shanghai: final results. J Natl Cancer Inst 2002, 94:1445–7.

38. Plesnicar A, Golicnik M, Fazarinc IK, et al. Attitudes of midwifery students towards teaching breast-self examination. Radiol Oncol 2010, 44:52–6.

39. Chalkidou K, Marquez P, Dhillon PK, et al. Evidence-informed frameworks for cost-effective cancer care and prevention in low, middle, and high-income countries. The Lancet. Oncology. 2014 Mar;15(3):e119-31.

40. Almutairi KM, Ahmad M, Vinluan JM, Almutairi A. Random Cross-Sectional Determination of the Level of Awareness Among Female Saudi Patients About Breast Cancer. Journal of cancer education. 2016 Mar;31(1):131-5.

41. DeSantis CE, Bray F, Ferlay J, et al. International Variation in Female Breast Cancer Incidence and Mortality Rates. Cancer epidemiology, biomarkers & prevention. 2015 Oct;24(10):1495-506.

For peer review only - http://bmjopen.bmj.com/site/about/guidelines.xhtml

Figure legends

Figure 1 Comparison of proportions of women aware of breast cancer symptoms between community-based and hospital-based studies

Figure 2 Proportions of sources of information of breast cancer for women

For peer review only

For peer review only - http://bmjopen.bmj.com/site/about/guidelines.xhtml

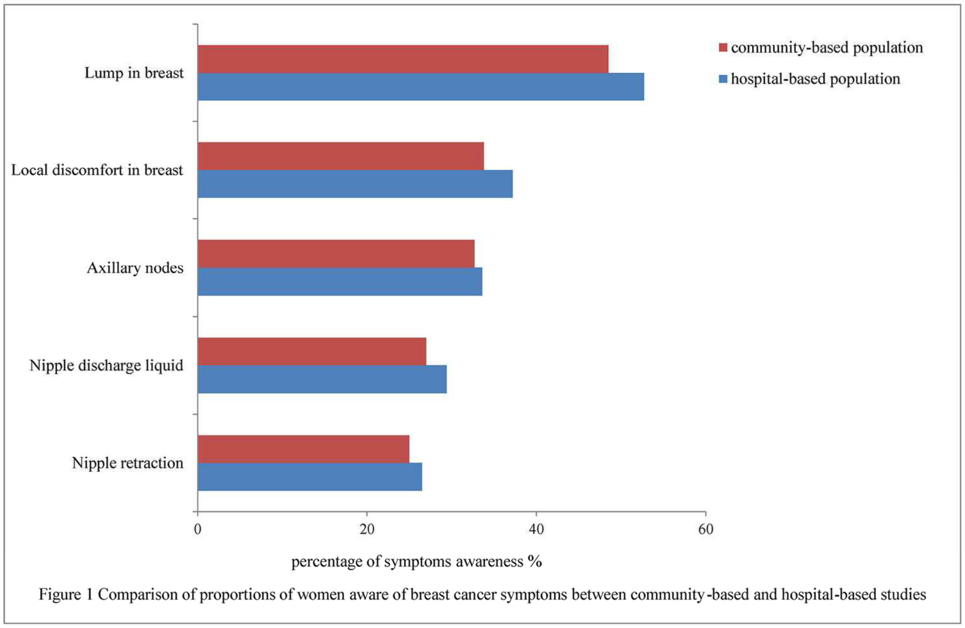

Figure 1 Comparison of proportions of women aware of breast cancer symptoms between community-based and hospital-based studies

152x101mm (300 x 300 DPI)

For peer review only - http://bmjopen.bmj.com/site/about/guidelines.xhtml

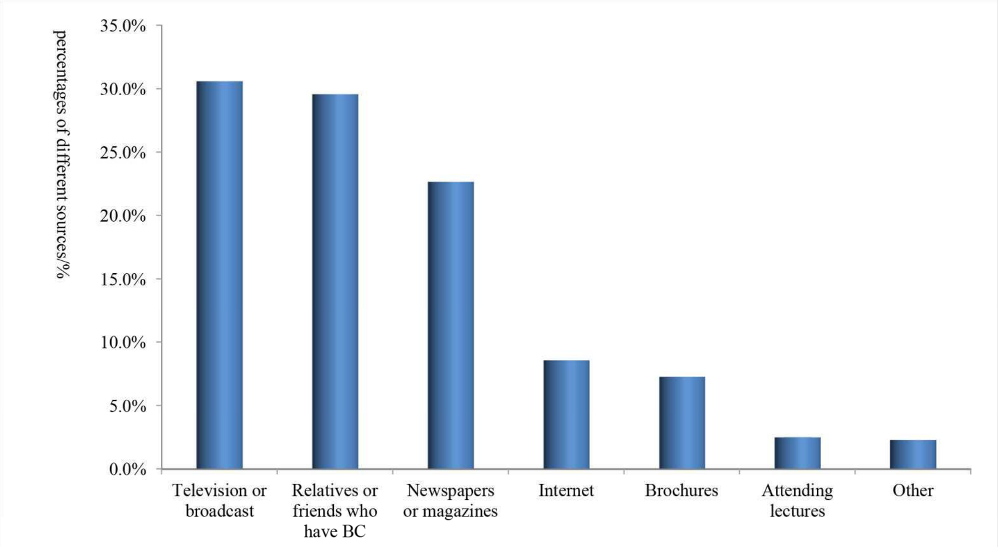

Figure 2 Proportions of sources of information of breast cancer for women

Figure 2 Proportions of sources of information of breast cancer for women

174x108mm (300 x 300 DPI)

STROBE Statement—Checklist of items that should be included in reports of ***case-control studies***

	Item No	Recommendation
**Title and abstract**	1	(*a*) Indicate the study's design with a commonly used term in the title or the abstract Page 2-3
		(*b*) Provide in the abstract an informative and balanced summary of what was done and what was found Page 2-3
**Introduction**		
Background/rationale	2	Explain the scientific background and rationale for the investigation being reported Page 4-5
Objectives	3	State specific objectives, including any prespecified hypotheses Page 5
**Methods**		
Study design	4	Present key elements of study design early in the paper Page 5
Setting	5	Describe the setting, locations, and relevant dates, including periods of recruitment, exposure, follow-up, and data collection Page 5-6
Participants	6	(*a*) Give the eligibility criteria, and the sources and methods of case ascertainment and control selection. Give the rationale for the choice of cases and controls Page 5-6
		(*b*) For matched studies, give matching criteria and the number of controls per case Page 5-6
Variables	7	Clearly define all outcomes, exposures, predictors, potential confounders, and effect modifiers. Give diagnostic criteria, if applicable Page 6-7
Data sources/ measurement	8*	For each variable of interest, give sources of data and details of methods of assessment (measurement). Describe comparability of assessment methods if there is more than one group Page 6-7
Bias	9	Describe any efforts to address potential sources of bias Page 7
Study size	10	Explain how the study size was arrived at Page 5
Quantitative variables	11	Explain how quantitative variables were handled in the analyses. If applicable, describe which groupings were chosen and why Page 6-7
Statistical methods	12	(*a*) Describe all statistical methods, including those used to control for confounding Page 8
		(*b*) Describe any methods used to examine subgroups and interactions Page 8
		(*c*) Explain how missing data were addressed Page 7
		(*d*) If applicable, explain how matching of cases and controls was addressed Page 5
		(*e*) Describe any sensitivity analyses Page 8
**Results**		
Participants	13*	(a) Report numbers of individuals at each stage of study—eg numbers potentially eligible, examined for eligibility, confirmed eligible, included in the study, completing follow-up, and analysed Page 8
		(b) Give reasons for non-participation at each stage
		(c) Consider use of a flow diagram
Descriptive data	14*	(a) Give characteristics of study participants (eg demographic, clinical, social) and information on exposures and potential confounders Page 8
		(b) Indicate number of participants with missing data for each variable of interest
Outcome data	15*	Report numbers in each exposure category, or summary measures of exposure Page8-11

For peer review only - http://bmjopen.bmj.com/site/about/guidelines.xhtml

Main results	16	(*a*) Give unadjusted estimates and, if applicable, confounder-adjusted estimates and their precision (eg, 95% confidence interval). Make clear which confounders were adjusted for and why they were included Page 8-15
		(*b*) Report category boundaries when continuous variables were categorized Page 8-15
		(*c*) If relevant, consider translating estimates of relative risk into absolute risk for a meaningful time period Page 16-17
Other analyses	17	Report other analyses done—eg analyses of subgroups and interactions, and sensitivity analyses
**Discussion**		
Key results	18	Summarise key results with reference to study objectives Page 17-21
Limitations	19	Discuss limitations of the study, taking into account sources of potential bias or imprecision. Discuss both direction and magnitude of any potential bias Page 17-21
Interpretation	20	Give a cautious overall interpretation of results considering objectives, limitations, multiplicity of analyses, results from similar studies, and other relevant evidence Page 21
Generalisability	21	Discuss the generalisability (external validity) of the study results Page 17
**Other information**		
Funding	22	Give the source of funding and the role of the funders for the present study and, if applicable, for the original study on which the present article is based Page 22

*Give information separately for cases and controls.

Note: An Explanation and Elaboration article discusses each checklist item and gives methodological background and published examples of transparent reporting. The STROBE checklist is best used in conjunction with this article (freely available on the Web sites of PLoS Medicine at http://www.plosmedicine.org/, Annals of Internal Medicine at http://www.annals.org/, and Epidemiology at http://www.epidem.com/). Information on the STROBE Initiative is available at http://www.strobe-statement.org.

For peer review only
